# Briarenols I—K, New Anti-inflammatory 8,17-Epoxybriaranes from the Octocoral *Briareum excavatum* (Briareidae)

**DOI:** 10.3390/molecules25061405

**Published:** 2020-03-19

**Authors:** Thanh-Hao Huynh, Lee-Shing Fang, Yu-Hsin Chen, Bo-Rong Peng, You-Ying Chen, Li-Guo Zheng, Yu-Jen Wu, Zhi-Hong Wen, Jih-Jung Chen, Tzu-Chi Lin, Ping-Jyun Sung

**Affiliations:** 1 Graduate Institute of Marine Biology, National Dong Hwa University, Pingtung 94450, Taiwan; haohuynh0108@gmail.com; 2 National Museum of Marine Biology and Aquarium, Pingtung 94450, Taiwan; kb5634@yahoo.com.tw (Y.-H.C.); pengpojung@gmail.com (B.-R.P.); zoeblack0108@gmail.com (Y.-Y.C.); t0919928409@gmail.com (L.-G.Z.); 3 Center for Environmental Toxin and Emerging-Contaminant Research, Cheng Shiu University, Kaohsiung 83347, Taiwan; lsfang@gcloud.csu.edu.tw; 4 Super Micro Mass Research and Technology Center, Cheng Shiu University, Kaohsiung 83347, Taiwan; 5 Department of Nursing, Meiho University, Pingtung 91202, Taiwan; x00002180@meiho.edu.tw; 6 Department of Marine Biotechnology and Resources, National Sun Yat-sen University, Kaohsiung 80424, Taiwan; wzh@mail.nsysu.edu.tw; 7 Faculty of Pharmacy, School of Pharmaceutical Sciences, National Yang-Ming University, Taipei 11221, Taiwan; chenjj@ym.edu.tw; 8 Department of Emergency Medicine, Antai Medical Care Corporation Antai Tian-Sheng Memorial Hospital, Pingtung 92842, Taiwan; 9 Chinese Medicine Research and Development Center, China Medical University Hospital, Taichung 40447, Taiwan; 10 Graduate Institute of Natural Products, Kaohsiung Medical University, Kaohsiung 80708, Taiwan

**Keywords:** *Briareum excavatum*, briarenol, briarane, anti-inflammatory, iNOS, COX-2

## Abstract

Five 8,17-epoxybriaranes, including three new compounds—briarenols I–K (**1**–**3**), along with two known analogues, briaexcavatolide P (**4**) and briaexcavatin P (**5**), were isolated from the octocoral *Briareum excavatum*. The structures of briaranes **1**–**3** were elucidated by spectroscopic methods, including 1D and 2D NMR studies and (+)-HRESIMS. Briarane **4** exerted inhibition effects on inducible nitric oxide synthase (iNOS) and cyclooxygenase-2 (COX-2) release from RAW 264.7.

## 1. Introduction

**Figure 1:**
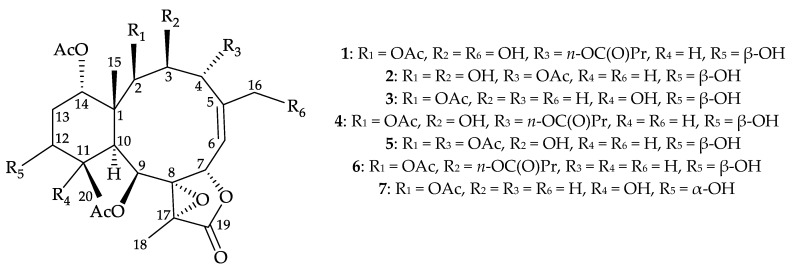
Structures of briarenols I–K (**1**–**3**), briaexcavatolide P (**4**), briaexcavatin P (**5**), excavatolide B (**6**), and briareolide B (**7**).

Octocorals of the genus *Briareum* (family Briareidae) [1,2,3,4] are proven to be the most important source to produce briarane-type diterpenoids [5]. The compounds of this type are only found in marine invertebrates, particularly in octocorals and demonstrated a wide spectrum of bioactivities, such as anti-inflammatory activity [6] and cytotoxicity [7]. In our continuing research into the chemical constituents of an octocoral *B. excavatum* (Nutting 1911), which was distributed extensively in the coral reefs of Taiwan, have resulted in isolation of three previously unreported 8,17-epoxybriaranes–briarenols I–K (**1**–**3**) along with two known analogues, briaexcavatolide P (**4**) [8] and briaexcavatin P (**5**) [9], (Figure 1). In the current study, the comprehensive workflow of isolation, structure determination, and anti-inflammatory activity evaluation, was implemented on briaranes **1**–**5**. 

## 2. Results and Discussion

**Figure 2:**
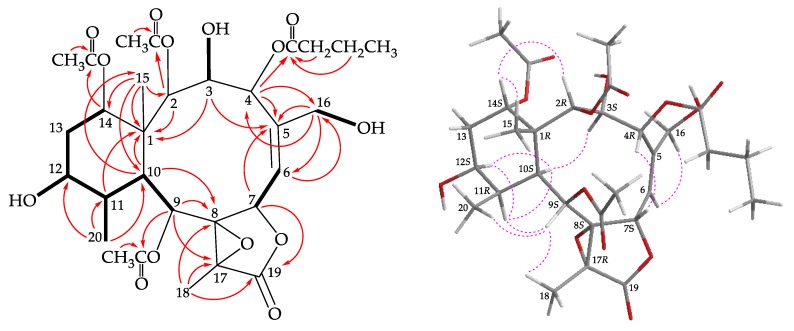
The COSY (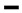) correlations, selective HMBC (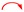), and protons with key NOESY correlations (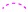) of **1**.

Briarenol I (**1**) was isolated as an amorphous powder and displayed a sodiated adduct ion at *m*/*z* 649.24677 in the (+)-HRESIMS, which indicated its molecular formula was C_30_H_42_O_14_ (calculated for C_30_H_42_O_14_ + Na, 649.24668; unsaturation degrees = 10). The IR spectrum revealed absorptions for hydroxy (ν_max_ 3524 cm^–1^), γ-lactone (ν_max_ 1783 cm^–1^), and ester carbonyl (ν_max_ 1736 cm^–1^) moieties. Resonances in the ^13^C NMR of **1** at δ_C_ 172.9, 172.3, 170.5, 170.0, and 170.0 (5 × C) supported the presence of a γ-lactone and four esters (Table 1). Three of the esters were identified as acetates by the presence of three methyl singlet resonances in the ^1^H NMR spectrum at δ_H_ 2.34, 2.15, and 2.08 (Table 2) and the remaining ester was found to be an *n*-butyroxy group based on ^1^H NMR studies, including a correlation spectroscopy (COSY) experiment, which revealed seven contiguous protons (δ_H_ 2.30, 2H, t, *J* = 7.2 Hz; 1.63, 2H, tq, *J* = 7.2 Hz; 0.95, 3H, t, *J* = 7.2 Hz). From the COSY spectrum (Figure 2), the proton sequences from H-6/H-7, H-9/H-10/H-11/H-12/H_2_-13/H-14, and H-11/H_3_-20 were established. The hydroxy proton signals at δ_H_ 4.30 (1H, d, *J* = 12.0 Hz), 1.49 (1H, d, *J* = 4.0 Hz), and 3.49 (1H, dd, *J* = 9.6, 4.4 Hz) were found to correlate with H-3 (δ_H_ 4.59, d, *J* = 12.0 Hz), H-12 (δ_H_ 4.10, m), and H_2_-16 (δ_H_ 4.35, 1H, dd, *J* = 13.6, 4.4 Hz; 4.04, 1H, dd, *J* = 13.6, 9.6 Hz), respectively. Thus, the hydroxy groups should be positioned at C-3, C-12, and C-16, respectively. Olefinic resonances in the ^13^C NMR at δ_C_ 125.5 (CH-6) and 142.0 (C-5) indicated the presence of a trisubstituted carbon–carbon double bond. On the basis of these data and the heteronuclear multiple bond correlation (HMBC) experiment (Figure 2), the connectivity from C-1 to C-14 was established. A hydroxymethyl group at C-5 was revealed by the HMBC between C-16 oxymethylene protons to C-4, C-5, and C-6. The C-15 methyl group at C-1 was confirmed by the HMBC between H_3_-15/C-1, C-2, C-10, C-14, and H-10/C-15. The *n*-butyrate positioned at C-4 was confirmed from the connectivity between H-4 (δ_H_ 6.14) and the carbonyl carbon of *n*-butyrate group (δ_C_ 172.3). HMBC from the oxymethine protons at δ_H_ 4.53 (H-2), 5.32 (H-9), and 4.88 (H-14) to the acetate carbonyls at δ_C_ 172.9, 170.0, and 170.0, placed the acetoxy groups on C-2, C-9, and C-14, respectively. Thirteen of the fourteen oxygen atoms in the molecular formula of 1 could be accounted for from the presence of a γ-lactone, four esters, and three hydroxy groups. The remaining oxygen atom had to be placed between C-8 and C-17 to form a tetrasubstituted epoxide based on the ^13^C NMR evidences at δ_C_ 70.8 (C-8) and 62.5 (C-17) and the ^1^H NMR chemical shift of a tertiary methyl at δ_H_ 1.66 (3H, s, H_3_-18).

The stereochemistry of **1** was deduced from an NOESY experiment (Figure 2) and biogenetic considerations. The NOE correlations of H-10/H-11, H-10/H-12, and H-11/H-12 indicated that these protons were situated on the same face of the structure and were assigned as the α protons since the C-15 methyl is the β-substituent at C-1. The NOE correlation between H_3_-15 and H-14 implied that H-14 had a β-orientation. H-3 exhibited a correlation with H-10, and, as well as a lack of coupling constants were detected between H-2/H-3 and H-3/H-4, indicating the dihedral angles between H-2/H-3 and H-3/H-4 were approximately 90° and the 2-acetoxy, 3-hydroxy, and 4-*n*-butyroxy groups were β-, β-, and α-oriented, respectively. A correlation from H-4 to H-7, suggested that H-7 was β-oriented. The *Z*-configuration of C-5/6 double bond was confirmed based on the fact that the C-6 olefinic proton (δ_H_ 5.53) correlated to one of the C-16 hydroxymethyl protons (δ_H_ 4.04). H-9 was found to correlate with H-11, H_3_-18, and H_3_-20. From a consideration of molecular model, H-9 was found to be reasonably close to H-11, H_3_-18, and H_3_-20, thus, H-9 should be placed on the α face, and Me-18 was β-oriented in the γ-lactone moiety, and the 8,17-epoxy group should be α-oriented. It was found that the NMR signals of **1** were similar to those of a known briarane, briaexcavatolide P (**4**) (Figure 1) [8], except that the signals corresponding to the Me-16 vinyl methyl in **4** were replaced by signals for a hydroxymethyl group in **1**. Additionally, as briaranes **1**–**5** were isolated along with a known briarane, excavatolide B (**6**) [6,10,11] from the same target organism, *B. excavatum*, and the absolute configuration of **6** was determined by a single-crystal X-ray diffraction analysis [6,11]. Therefore, on biogenetic grounds to assume that briaranes **1**–**5** had the same absolute stereochemistry as that of **6**, tentatively, and the configurations of stereogenic carbons of **1** were determined as 1*R*,2*R*,3*S*,4*R*,7*S*,8*S*, 9*S*,10*S*,11*R*,12*S*,14*S*, and 17*R* (Supplementary Materials, Figures S1–S10).

Briarenol J (**2**) had a molecular formula C_26_H_36_O_12_ by its (+)-HRESIMS at *m*/*z* 563.21007 (calculated for C_26_H_36_O_12_ + Na, 563.20990). The IR spectrum showed bands at 3483, 1779, and 1727 cm^−1^, consistent with the presence of hydroxy, γ-lactone, and ester groups, respectively, in **2**. From the ^13^C and DEPT data (Table 2), one trisubstituted double bond was deduced from the signals of two carbons at δ_C_ 139.3 (C-5) and 124.3 (CH-6). A methyl-containing tetrasubstituted epoxy group was confirmed from the signals of two oxygenated quaternary carbons at δ_C_ 69.9 (C-8) and 61.8 (C-17), and from the chemical shift of a tertiary methyl (δ_H_ 1.66, 3H, s; δ_C_ 10.3, CH_3_-18; Table 1 and Table 2). Four carbonyl resonances at δ_C_ 170.9, 170.0, 169.5, and 169.2 in the ^13^C spectrum confirmed the presence of a γ-lactone and three esters. All the esters were identified as acetates by the presence of three methyl singlet resonances in the ^1^H NMR spectrum at δ_H_ 2.32, 2.16, and 2.14, respectively.

**Figure 3:**
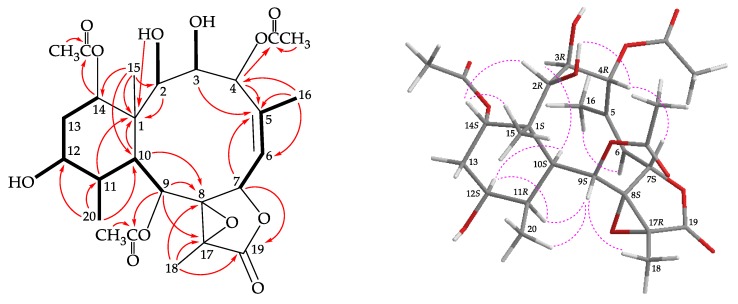
The COSY (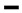) correlations, selective HMBC (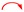), and protons with key NOESY correlations (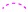) of **2**.

Coupling constants information in the COSY spectrum of **2** enabled identification of H-6/H-7, H-9/H-10/H-11/H-12/H_2_-13/H-14, H-11/H_3_-20, and H-6/H_3_-16 (by allylic coupling; Figure 3), these data, together with the HMBC experiment (Figure 3), the molecular framework of **2** could be established. The HMBC also indicated that the acetoxy groups should be attached at C-4, C-9, and C-14, respectively. Thus, the remaining hydroxy groups have to be positioned at C-2, C-3, and C-12, as indicated by the COSY correlations between H-2/OH-2, H-3/OH-3, and H-12/OH-12.

The stereochemistry of **2** was elucidated from the NOE interactions observed in a NOESY experiment (Figure 3) and by the vicinal ^1^H−^1^H coupling constant analysis. In the NOESY spectrum, correlations were observed between H-10 with H-3 and H-12; and H-12 correlated with H-11, indicating that these protons should be α-oriented. H-14 gave a correlation with H_3_-15, confirming the β-orientation for this proton. H-2 showed a correlation with H-14, and a lack of coupling constant was detected between H-2/H-3, indicating the dihedral angle between H-2/H-3 is approximately 90° and the 2-hydroxy group was β-oriented. H-4 exhibited correlations with H-7 and 2-hydroxy proton, confirming the β-orientations for H-4 and H-7. H-9 was found to show correlations with H-11, H_3_-18, and H_3_-20, and from molecular models, H-9 and H_3_-18 should be placed on the α- and β-face, respectively. The *Z*-configuration of C-5/C-6 double bond was elucidated by a correlation between H-6 and H_3_-16. The NMR data of **2** were found to be similar to those of a known briarane, briaexcavatin P (**5**) [9]. It was found that the 2-acetoxy substituent in **5** was replaced by a hydroxy group in **2**. By comparison of the proton and carbon chemical shifts, coupling constants, NOESY correlations, and rotation value of **2** with those of **5**, the stereochemistry of **2** was confirmed to be the same as that of **5**, and the configurations of the stereogenic centers of **2** were assigned as 1*S*,2*R*,3*R*,4*R*, 7*S*,8*S*,9*S*,10*S*,11*R*,12*S*,14*S*, and 17*R* (Supplementary Materials, Figures S11–S20). 

**Figure 4:**
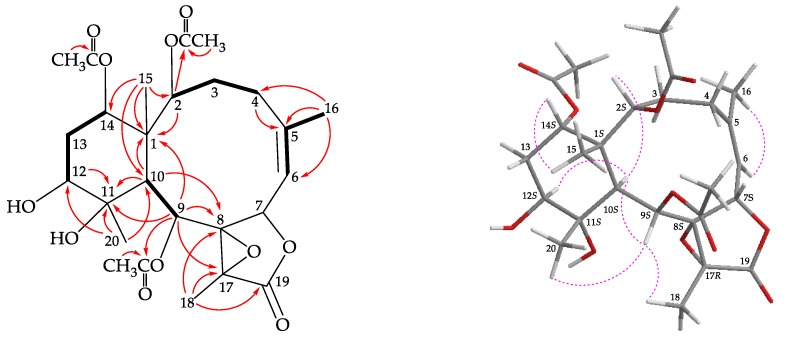
The COSY (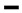) correlations, selective HMBC (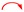), and protons with key NOESY correlations (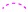) of **3**.

Briarane **3** (briarenol K) was found to have a molecular formula of C_26_H_36_O_11_ based on its (+)-HRESIMS peak at *m*/*z* 547.21514 (calculated for C_26_H_36_O_11_ + Na, 547.21498). Its absorption peaks in the IR spectrum showed ester carbonyl, γ-lactone, and broad OH stretching at 1739, 1780, and 3468 cm^−1^, respectively. The ^13^C NMR spectrum indicated that three esters and a γ-lactone were present, as carbonyl resonances were observed at δ_C_ 168.1, 170.2, 170.4, and 170.4, respectively (Table 1). The ^1^H NMR data also indicated that presence of three acetate methyls at δ_H_ 2.22, 2.03, and 2.00 (each 3H × s; Table 2). It was found that the spectroscopic data of **3** were similar to those of a known briarane, briareolide B (**7**) [12]; however, by comparison of the ^1^H and ^13^C NMR chemical shifts of CH-12 oxymethine (δ_H_ 3.72, 1H, dd, *J* = 12.4, 4.8 Hz; δ_C_ 73.4), CH_2_-13 sp^3^ methylene (δ_H_ 1.67, 1H, m; 2.04, 1H, m; δ_C_ 30.2), C-11 oxygenated quaternary carbon (δ_C_ 78.2), and Me-20 tertiary methyl (δ_H_ 1.15, 3H, s; δ_C_ 16.9) of **3** with those of **7** (δ_H_ 3.56, 1H, m; δ_C_ 73.9, CH-12; δ_H_ 2.03, 1H, m; 2.12, 1H, m; δ_C_ 27.6, CH_2_-13; δ_C_ 74.7, C-11; δ_H_ 1.16, 3H, s; δ_C_ 22.5, Me-20) [12] showed that the hydroxy group at C-12 in **3** was β-oriented. The locations of the functional groups were further confirmed by other HMBC and COSY correlations (Figure 4), and hence briarenol K was assigned the structure of **3**. The NOESY spectrum exhibited a correlation from H-10 to H-12, further supporting that H-12 was α-oriented and the stereogenic centers of **3** were assigned as 1*S*,2*S*,7*S*,8*S*,9*S*,10*S*,11*S*,12*S*,14*S*, and 17*R*, by the correlations observed in a NOESY spectrum (Figure 4) and this compound was found to be the 12-epimer of briareolide B (**7**) [12] (Supplementary Materials, Figures S21–S30).

**Figure 5:**
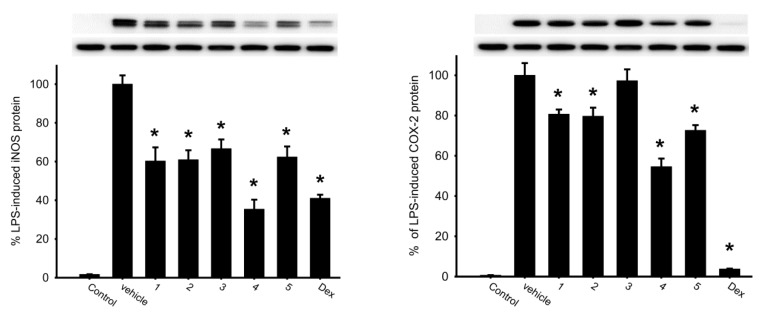
Western blotting showed that briarane **4** downregulated the expression of iNOS and COX-2. Data were normalized to the cells treated with LPS only, and cells treated with dexamethasone (Dex; 10 µM) were used as a positive control. Data are expressed as the mean ± SEM (*n* = 2~4). * Significantly different from cells treated with LPS (*p* < 0.05).

The inhibition effects of briaranes **1**–**5** on the release of inducible nitric oxide synthase (iNOS) and cyclooxygenase-2 (COX-2) protein from lipopolysaccharides (LPS)-stimulated RAW 264.7 were assessed. The results showed that briarane **4** reduced the release of iNOS and COX-2 to 35.37% and 54.61% at a concentration of 10 µM, respectively (Figure 5 and Table 3). Briarane **1** was found to be weaker than those of **4** in term of reducing the expression of iNOS and COX-2, indicating that the hydroxy group at C-16 in **1** reduced the activity.

## 3. Materials and Methods

### 3.1. General Experimental Procedures

Optical rotation values were measured using a Jasco P-1010 digital polarimeter (Japan Spectroscopic, Tokyo, Japan). IR spectra were measured on a Thermo Scientific Nicolet iS5 FT-IR spectrophotometer (Waltham, MA, USA). NMR spectra were taken on a Jeol Resonance ECZ 400 S NMR spectrometer (Tokyo, Japan), using the residual CHCl_3_ signal (δ_H_ 7.26 ppm) and CDCl_3_ (δ_C_ 77.1 ppm) as the internal standard for ^1^H and ^13^C NMR, respectively; coupling constants (*J*) are presented in Hz. ESIMS and HRESIMS were recorded using a Bruker 7 Tesla solariX FTMS system. Column chromatography was carried out with silica gel (230–400 mesh, Merck, Darmstadt, Germany). TLC was performed on plates precoated with Kieselgel 60 F_254_ (0.25-mm-thick, Merck, Darmstadt, Germany), then sprayed with 10% H_2_SO_4_ solution followed by heating to visualize the spots. Normal-phase HPLC (NP-HPLC) was performed using a system comprised of a Hitachi L-7100 pump (Tokyo, Japan) and a Rheodyne 7725i injection port (Rohnert Park, CA, USA). Reverse-phase HPLC (RP-HPLC) was performed using a system comprised of a Hitachi L-2130 pump (Tokyo, Japan), a Hitachi L-2455 photodiode array detector (Tokyo, Japan), and a Rheodyne 7725i injection port (Rohnert Park, CA, USA). A semipreparative normal-phase column (YMC-Pack SIL, S-5 µm, 250 mm × 20 mm, Sigma-Aldrich, St. Louis, MO, USA) was used for NP-HPLC. A semipreparative reverse-phase column (Luna, 5 µm, C18(2) 100 Å, AXIA Packed, 250 mm × 21.2 mm; Phenomenex, Torrance, CA, USA) was used for RP-HPLC.

### 3.2. Animal Material

Specimens of *B. excavatum* were collected in June 2017 by hand with self-contained underwater breathing apparatus (SCUBA*)* divers off the coast of Lanyu Island (Orchid Island), Taiwan. The samples were then stored in a –20 °C freezer until extraction. A voucher specimen was deposited in the National Museum of Marine Biology and Aquarium, Taiwan (NMMBA-TW-SC-2017-418). Identification of the species of this organism was performed by comparison as described in previous publications [1,2,3,4].

### 3.3. Extraction and Isolation

The freeze-dried and sliced bodies (wet/dry weight = 1344/568 g) of the specimen were extracted with supercritical CO_2_ to give 58.9 g of extract. Partial extract (36.4 g) was then applied on silica gel column and eluted with gradients of *n*-hexane/EtOAc to furnish fractions A−K. Fraction F was purified by NP-HPLC using a mixture of *n*-hexane/acetone (4:1) to yield fractions F1−F13. Fraction F6 was repurified by RP-HPLC, using a mixture of MeOH/H_2_O (60:40; at a flow rate = 4 mL/min) to afford **4** (6.7 mg). Fraction G was separated by NP-HPLC, using a mixture of *n*-hexane/acetone (3:1) to yield fractions G1−G12. Fractions G6 and G7 were repurified by RP-HPLC using a mixture of MeOH/H_2_O (60:40; at a flow rate = 4.0 mL/min) to afford **5** (1.3 mg) and **3** (1.0 mg), respectively. Fraction H was separated by NP-HPLC using a mixture of *n*-hexane and acetone (3:1) to yield fractions H1−H18. Fractions H12 and H15 were repurified by RP-HPLC, using a mixture of MeOH/ H_2_O (60:40; at a flow rate = 4.0 mL/min) to afford **2** (2.1 mg) and **1** (0.6 mg), respectively.

Briarenol I (**1**): Amorphous powder; ${\left[\alpha \right]}_{D}^{22}$ + 207 (*c* 0.03, CHCl_3_), IR (ATR) ν_max_ 3524, 1783, 1736, 1222, 891 cm^−1^; ^13^C (100 MHz, CDCl_3_) and ^1^H (400 MHz, CDCl_3_) NMR data (see Table 1 and Table 2); ESIMS: *m*/*z* 649 [M + Na]^+^; HRESIMS *m*/*z* 649.24677 (calculated for C_30_H_42_O_14_ + Na, 649.24668).

Briarenol J (**2**): Amorphous powder; ${\left[\alpha \right]}_{D}^{26}$ + 140 (*c* 0.08, CHCl_3_), IR (ATR) ν_max_ 3483, 1779, 1727, 1220, 890 cm^−1^; ^13^C (100 MHz, CDCl_3_) and ^1^H (400 MHz, CDCl_3_) NMR data (see Table 1 and Table 2); ESIMS: *m*/*z* 563 [M + Na]^+^; HRESIMS *m*/*z* 563.21007 (calculated for C_26_H_36_O_12_ + Na, 563.20990).

Briarenol K (**3**): Amorphous powder; ${\left[\alpha \right]}_{D}^{23}$ + 37 (*c* 0.06, CHCl_3_), IR (ATR) ν_max_ 3468, 1780, 1739, 1255, 892 cm^−1^; ^13^C (100 MHz, CDCl_3_) and ^1^H (400 MHz, CDCl_3_) NMR data (see Table 1 and Table 2); ESIMS: *m*/*z* 547 [M + Na]^+^; HRESIMS *m*/*z* 547.21514 (calculated for C_26_H_36_O_11_ + Na, 547.21498).

Briaexcavatolide P (**4**): Amorphous powder; ${\left[\alpha \right]}_{D}^{24}$ + 182 (*c* 0.3, CHCl_3_) (ref. [8], [α]27D + 167 (*c* 1.0, CHCl_3_)), IR (ATR) ν_max_ 3513, 1783, 1731, 1218, 889 cm^−1^; ^1^H and ^13^C NMR data were found to be in agreement with previous study [8]; ESIMS: *m*/*z* 633 [M + Na]^+^.

Briaexcavatin P (**5**): Amorphous powder; ${\left[\alpha \right]}_{D}^{23}$ + 134 (*c* 0.05, CHCl_3_) (ref. [9], ${\left[\alpha \right]}_{D}^{25}$ + 198 (*c* 0.08, CHCl_3_)), IR (ATR) ν_max_ 3503, 1785, 1735, 1240, 889 cm^−1^; ^1^H and ^13^C NMR data were found to be in agreement with previous study [9]; ESIMS: *m*/*z* 605 [M + Na]^+^.

### 3.4. In Vitro Anti-inflammatory Assay

The proinflammatory suppression assay was employed to assess the activities of the isolated compounds **1**–**5** against the release of iNOS and COX-2 from macrophage cells as the literature reported [13,14,15].

## 4. Conclusions

*B. excavatum* was demonstrated to have a wide structural diversity of briarane-type diterpenoids that possessed various pharmacological properties, especially in anti-inflammatory activity. In our continued study on *B. excavatum*, three previously unreported briaranes, briarenols I–K (**1**–**3**), along with the known analogues, briaexcavatolide P (**4**) and briaexcavatin P (**5**), were isolated. In the present study, the anti-inflammatory activity of **1**–**5** was assessed using inhibition of pro-inflammatory iNOS and COX-2 release from macrophages. The results indicated that briaexcavatolide P (**4**) showed the most potent suppressive effect on iNOS release.

## Figures and Tables

**Table 1:** ^13^C NMR (δ_C_ 100 MHz, CDCl_3_) data for briaranes **1**–**3**.

Position	1	2	3
1	43.3, C ^a^	43.7, C	47.7, C
2	87.4, CH	85.6, CH	74.9, CH
3	73.1, CH	73.6, CH	31.6, CH_2_
4	66.0, CH	65.9, CH	28.4, CH_2_
5	142.0, C	139.3, C	144.8, C
6	125.5, CH	124.3, CH	118.4, CH
7	74.1, CH	74.0, CH	74.9, CH
8	70.8, C	69.9, C	70.8, C
9	66.2, CH	67.1, CH	67.4, CH
10	40.5, CH	41.4, CH	49.0, CH
11	37.2, CH	36.4, CH	78.2, C
12	66.6, CH	67.0, CH	73.4, CH
13	30.2, CH_2_	30.4, CH_2_	30.2, CH_2_
14	80.5, CH	80.1, CH	74.8, CH
15	18.6, CH_3_	19.1, CH_3_	14.3, CH_3_
16	62.5, CH_2_	16.8, CH_3_	27.2, CH_3_
17	62.5, C	61.8, C	66.5, C
18	10.3, CH_3_	10.3, CH_3_	10.4, CH_3_
19	170.5, C	170.9, C	170.4, C
20	8.9, CH_3_	8.7, CH_3_	16.9, CH_3_
OAc-2	172.9, C		170.2, C
	21.2, CH_3_		21.2, CH_3_
OAc-4		169.5, C	
		21.0, CH_3_	
OAc-9	170.0, C	169.2, C	168.1, C
	21.5, CH_3_	21.1, CH_3_	21.5, CH_3_
OAc-14	170.0, C	170.0, C	170.4, C
	21.2, CH_3_	21.0, CH_3_	21.3, CH_3_
*n*-OC(O)Pr-4	172.3, C		
	35.9, CH_2_		
	18.2, CH_2_		
	13.7, CH_3_		

^a^ Multiplicity deduced by DEPT and HSQC spectra.

**Table 2:** ^1^H NMR (δ_H_, 400 MHz in CDCl_3_) data (*J* in Hz) for briaranes **1**–**3**.

Position	1	2	3
2	4.53 s	3.45 d (10.4)	5.13 d (8.4)
3α/β	4.59 d (12.0)	4.27 d (10.4)	1.67 m; 2.60 ddd (16.0, 14.8, 6.0)
4/4′	6.14 s	6.05 d (1.2)	2.48 br d (16.0); 1.90 m
6	5.53 d (6.0)	5.29 dq (6.4, 1.6)	5.19 s
7	5.62 d (6.0)	5.71 d (6.4)	5.19 s
9	5.32 d (8.8)	5.26 d (9.2)	5.78 d (1.2)
10	2.64 dd (8.8, 4.8)	2.55 dd (9.2, 5.2)	2.14 br s
11	2.41 m	2.47 m	
12	4.10 m	4.05 m	3.72 dd (12.4, 4.8)
13α/β	1.75 m; 2.01 m	1.69 m; 2.00 m	1.67 m; 2.04 m
14	4.88 dd (2.8, 2.8)	4.92 dd (2.8, 2.8)	4.79 dd (2.4, 2.0)
15	0.99 s	0.99 br s	1.21 s
16a/b	4.35 dd (13.6, 4.4); 4.04 dd (13.6, 9.6)	1.89 br s	1.99 s
18	1.66 s	1.66 s	1.77 s
20	1.12 d (6.8)	1.07 d (7.2)	1.15 s
OH-2		2.79 d (10.4)	
OH-3	4.30 d (12.0)	2.87 d (10.4)	
OH-12	1.49 d (4.0)	1.43 d (4.0)	-
OH-16	3.49 dd (9.6, 4.4)		
OAc-2	2.08 s		2.00 s
OAc-4		2.14 s	
OAc-9	2.34 s	2.32 s	2.22 s
OAc-14	2.15 s	2.16 s	2.03 s
*n*-OC(O)Pr-4	0.95 t (7.2)		
	1.63 tq (7.2)		
	2.30 t (7.2)		

**Table 3:** Effects of briaranes **1**–**5** on LPS-induced pro-inflammatory iNOS and COX-2 protein expression in macrophages.

	iNOS	COX-2	β-Actin	
	Expression (% of LPS)	Expression (% of LPS)	Expression (% of LPS)	*n*
Negative Control	1.71 ± 0.13	0.62 ± 0.09	120.48 ± 1.28	2
LPS	100.00 ± 4.53	100.00 ± 6.05	100.00 ± 3.09	4
1	60.27 ± 7.05	80.63 ± 2.32	100.29 ± 2.46	4
2	60.94 ± 4.89	79.65 ± 4.27	98.29 ± 3.35	4
3	66.64 ± 4.79	97.28 ± 5.66	100.49 ± 6.44	4
4	35.37 ± 4.94	54.61 ± 4.03	104.56 ± 2.83	4
5	62.36 ± 5.42	72.63 ± 2.6	104.79 ± 2.76	4
Dexamethasone	41.00 ± 2.63	3.73 ± 0.35	104.24 ± 5.82	2

Data were normalized to those of cells treated with LPS alone, and cells treated with dexamethasone were used as a positive control. Data are expressed as the mean ± SEM (*n* = 2–4).

## Supplementary Materials

The Supplementary Materials are available online. ESIMS, HRESIMS, IR, 1D and 2D NMR spectra of new compounds **1**–**3**.
