# Supplementary material for: Briarenols I—K, New Anti-inflammatory 8,17-Epoxybriaranes from the Octocoral Briareum excavatum (Briareidae)
Source: Molecules. 2020 Mar 19;25(6):1405. doi: 10.3390/molecules25061405 (PMC7144368; doi:10.3390/molecules25061405)
Supplement: Supplementary file 1 [file molecules-25-01405-s001.pdf]

molecules

Supplementary

Briarenols I—K, New Anti-inflammatory 8,17-Epoxybriaranes from the Octocoral *Briareum excavatum* (Briareidae)

Thanh-Hao Huynh [1,2], Lee-Shing Fang [3,4], Yu-Hsin Chen [2], Bo-Rong Peng [2] You-Ying Chen [2], Li-Guo Zheng [2], Yu-Jen Wu [5], Zhi-Hong Wen [6], Jih-Jung Chen [7], Tzu-Chi Lin [8,*] and Ping-Jyun Sung [1,2,6,9,10,*]

[1] Graduate Institute of Marine Biology, National Dong Hwa University, Pingtung 94450, Taiwan; haohuynh0108@gmail.com (T.-H.H.)
[2] National Museum of Marine Biology and Aquarium, Pingtung 94450, Taiwan; kb5634@yahoo.com.tw (Y.-H.C.); pengpojung@gmail.com (B.-R.P.); zoeblack0108@gmail.com (Y.-Y.C.); t0919928409@gmail.com (L.-G.Z.)
[3] Center for Environmental Toxin and Emerging-Contaminant Research, Cheng Shiu University, Kaohsiung 83347, Taiwan; lsfang@gcloud.csu.edu.tw (L.-S.F.)
[4] Super Micro Mass Research and Technology Center, Cheng Shiu University, Kaohsiung 83347, Taiwan
[5] Department of Nursing, Meiho University, Pingtung 91202, Taiwan; x00002180@meiho.edu.tw (Y.-J.W.)
[6] Department of Marine Biotechnology and Resources, National Sun Yat-sen University, Kaohsiung 80424, Taiwan; wzh@mail.nsysu.edu.tw (Z.-H.W.)
[7] Faculty of Pharmacy, School of Pharmaceutical Sciences, National Yang-Ming University, Taipei 11221, Taiwan; chenjj@ym.edu.tw (J.-J.C.)
[8] Department of Emergency Medicine, Antai Medical Care Corporation Antai Tian-Sheng Memorial Hospital, Pingtung 92842, Taiwan
[9] Chinese Medicine Research and Development Center, China Medical University Hospital, Taichung 40447, Taiwan
[10] Graduate Institute of Natural Products, Kaohsiung Medical University, Kaohsiung 80708, Taiwan
* Correspondence: Bryan1110@hotmail.com (T.-Z.L.); pjsung@nmmba.gov.tw (P.-J.S.); Tel.: +886-8-882-5037 (T.-Z.L.); Fax: +886-8-882-5087 (P.-J.S.)

Table of Contents

Figure S1. ESIMS spectrum of compound 1. 3

Figure S2. HRESIMS spectrum of compound **1.** 4

Figure S3. IR spectrum of compound **1.** 4

Figure S4. ^{1}H NMR spectrum (400 MHz) of compound **1** in $CDCl_3$. 5

Figure S5. ^{13}C NMR spectrum (100 MHz) of compound **1** in $CDCl_3$. 5

Figure S6. DEPT spectrum (100 MHz) of compound **1** in $CDCl_3$. 6

Figure S7. HSQC spectrum of compound **1** in $CDCl_3$. 6

Figure S8. HMBC spectrum of compound **1** in $CDCl_3$. 7

Figure S9. ^{1}H-^{1}H COSY spectrum of compound **1** in $CDCl_3$. 7

Figure S10. NOESY spectrum of compound **1** in $CDCl_3$. 8

Figure S11. ESIMS spectrum of compound **2.** 9

Figure S12. HRESIMS spectrum of compound **2.** 10

Figure S13. IR spectrum of compound **2.** 10

Figure S14. 1H NMR spectrum (400 MHz) of compound 2 in CDCl3 11

Figure S15. ^{13}C NMR spectrum (100 MHz) of compound **2** in $CDCl_3$. 11

Figure S16. DEPT spectrum (100 MHz) of compound **2** in $CDCl_3$. 12

Figure S17. HSQC spectrum of compound **2** in $CDCl_3$. 12

Figure S18. HMBC spectrum of compound **2** in $CDCl_3$. 13

Figure S19. ^{1}H-^{1}H COSY spectrum of compound **2** in $CDCl_3$. 13

Figure S20. NOESY spectrum of compound **2** in $CDCl_3$. 14

Figure S21. ESIMS spectrum of compound **3.** 15

Figure S22. HRESIMS spectrum of compound **3.** 16

Figure S23. IR spectrum of compound **3.** 16

Figure S24. ^{1}H NMR spectrum (400 MHz) of compound **3** in $CDCl_3$. 17

Figure S25. ^{13}C NMR spectrum (100 MHz) of compound **3** in $CDCl_3$. 17

Figure S26. DEPT spectrum (100 MHz) of compound **3** in $CDCl_3$. 18

Figure 27. HSQC spectrum of compound **3** in $CDCl_3$. 18

Figure S28. HMBC spectrum of compound **3** in $CDCl_3$. 19

Figure 29. ^{1}H-^{1}H COSY spectrum of compound **3** in $CDCl_3$. 19

Figure S30. NOESY spectrum of compound **3** in $CDCl_3$. 20

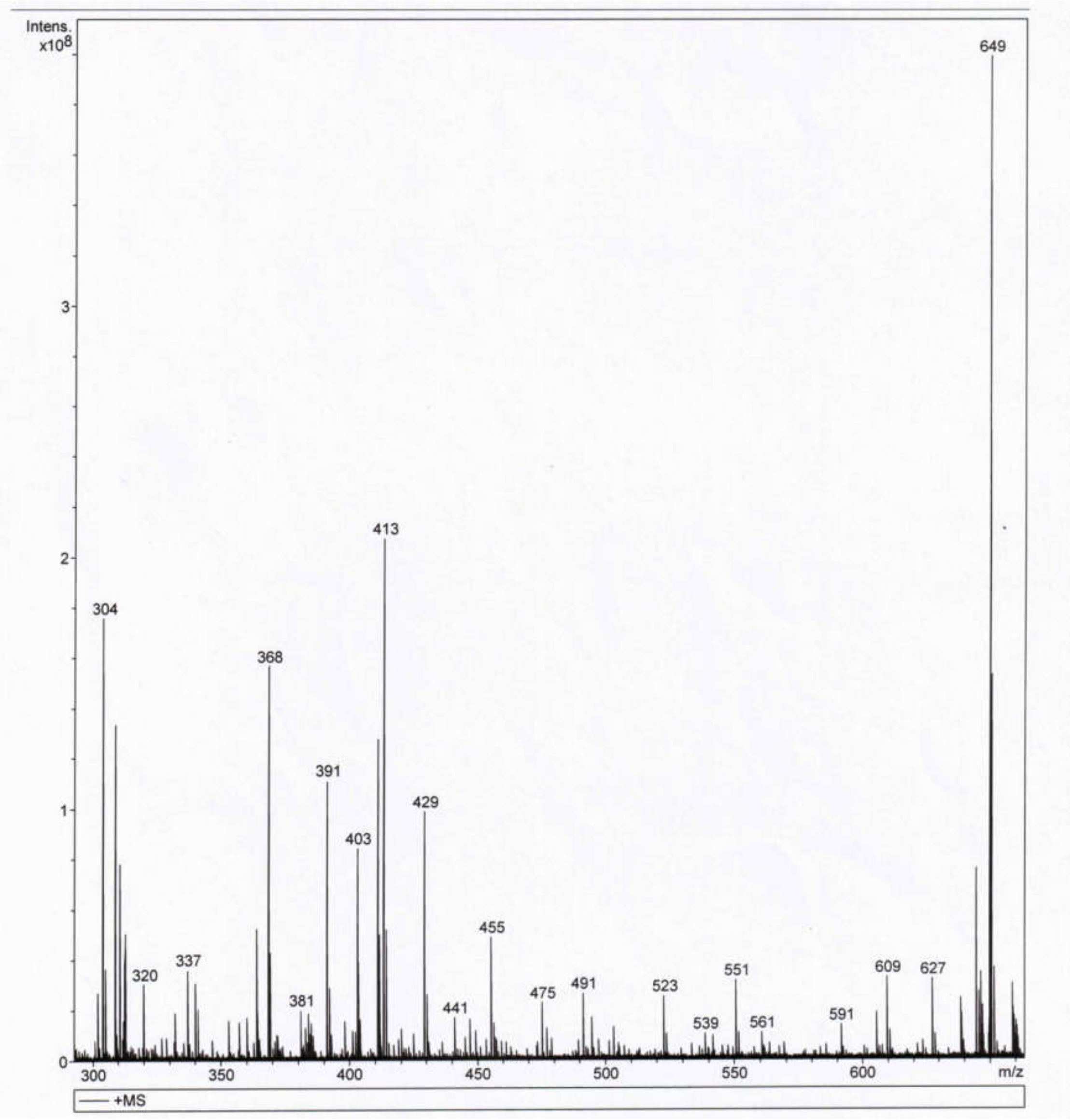

Figure S1. ESIMS spectrum of compound 1.

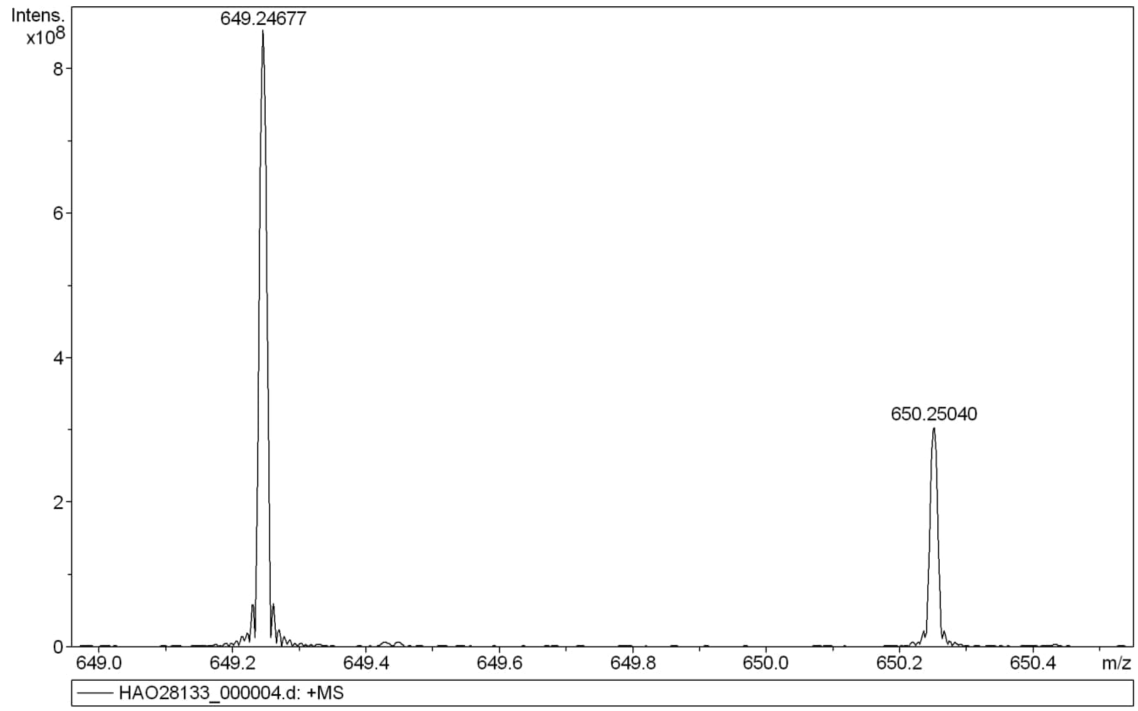

Meas. m/z	#	Formula	Score	m/z	err [mDa]	err [ppm]	mSigma	rdb	e⁻ Conf	N-Rule
649.24677	1	C 30 H 42 Na O 14	100.00	649.24668	-0.10	-0.15	11.8	9.5	even	ok

Figure S2. HRESIMS spectrum of compound **1.**

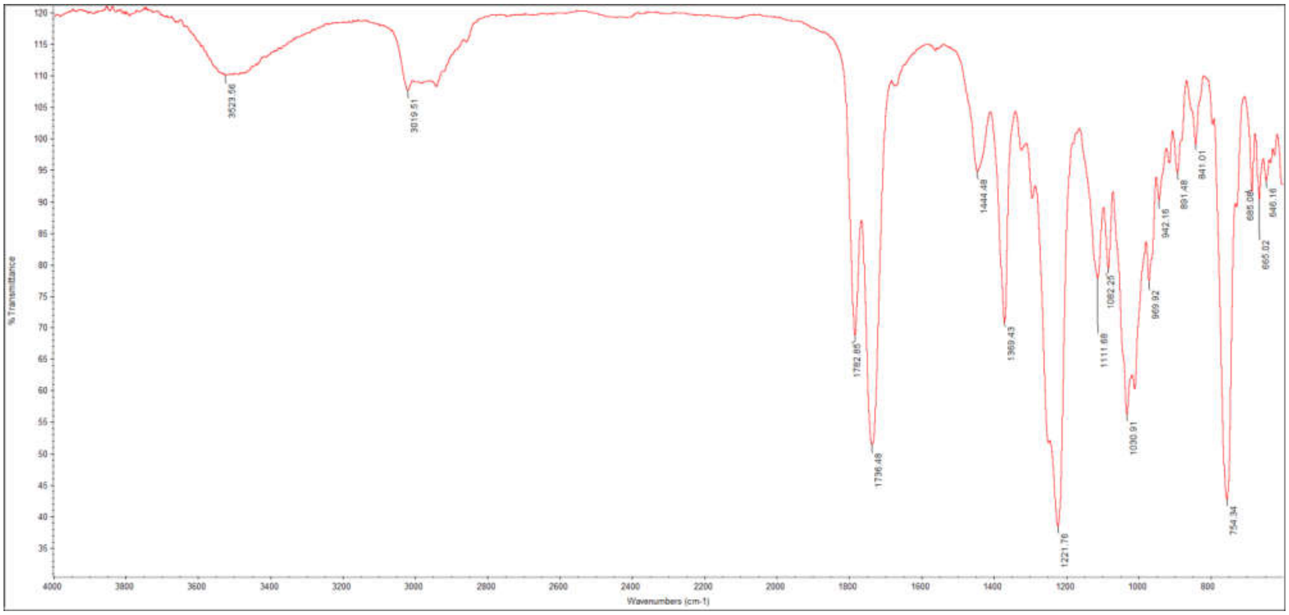

Figure S3. IR spectrum of compound **1.**

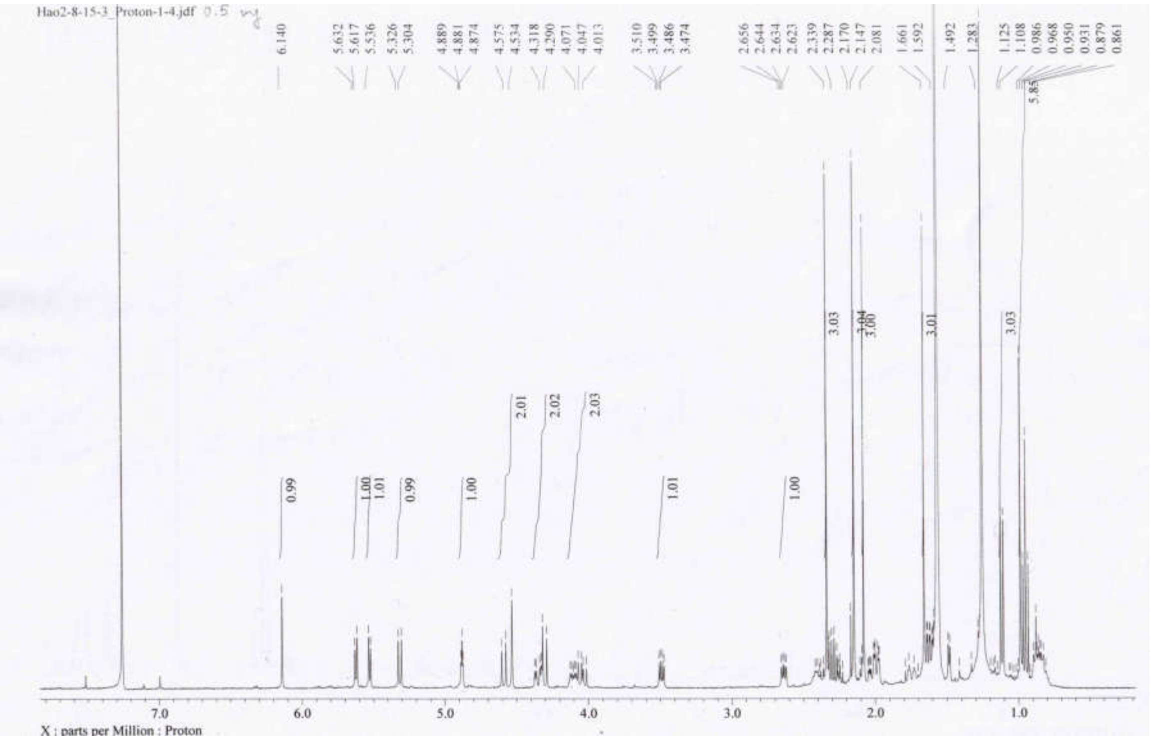

Figure S4. ^{1}H NMR spectrum (400 MHz) of compound **1** in $CDCl_3$.

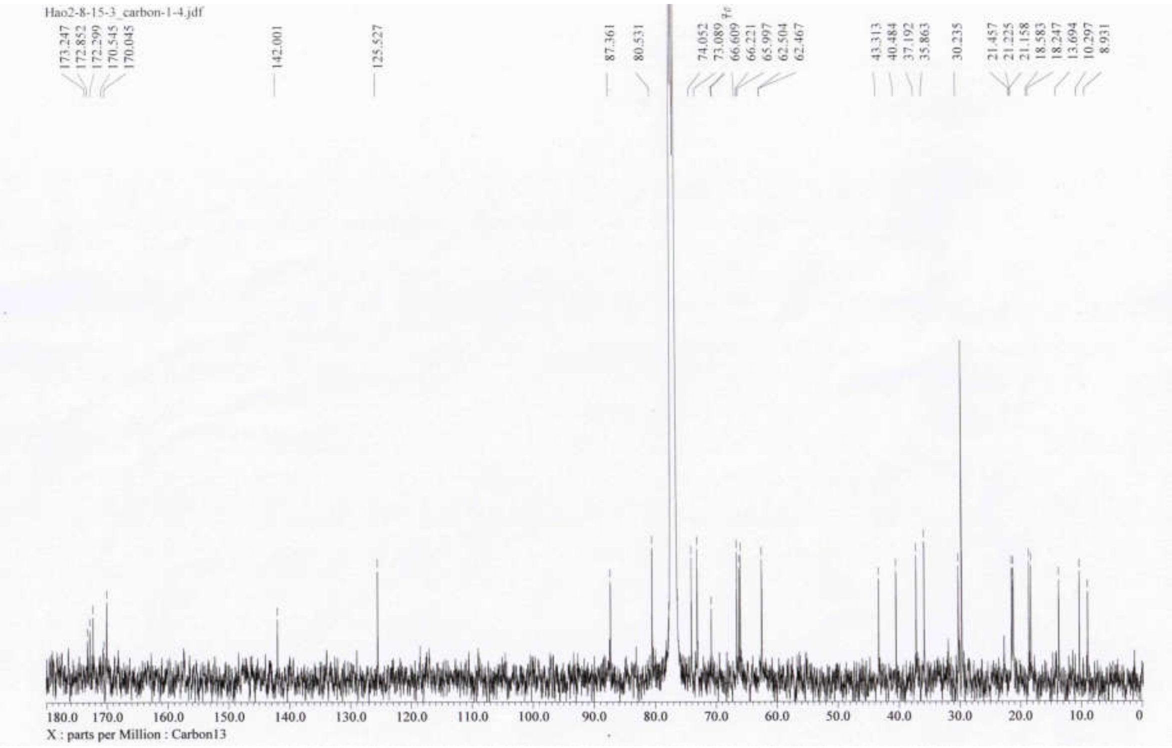

Figure S5. ^{13}C NMR spectrum (100 MHz) of compound **1** in $CDCl_3$.

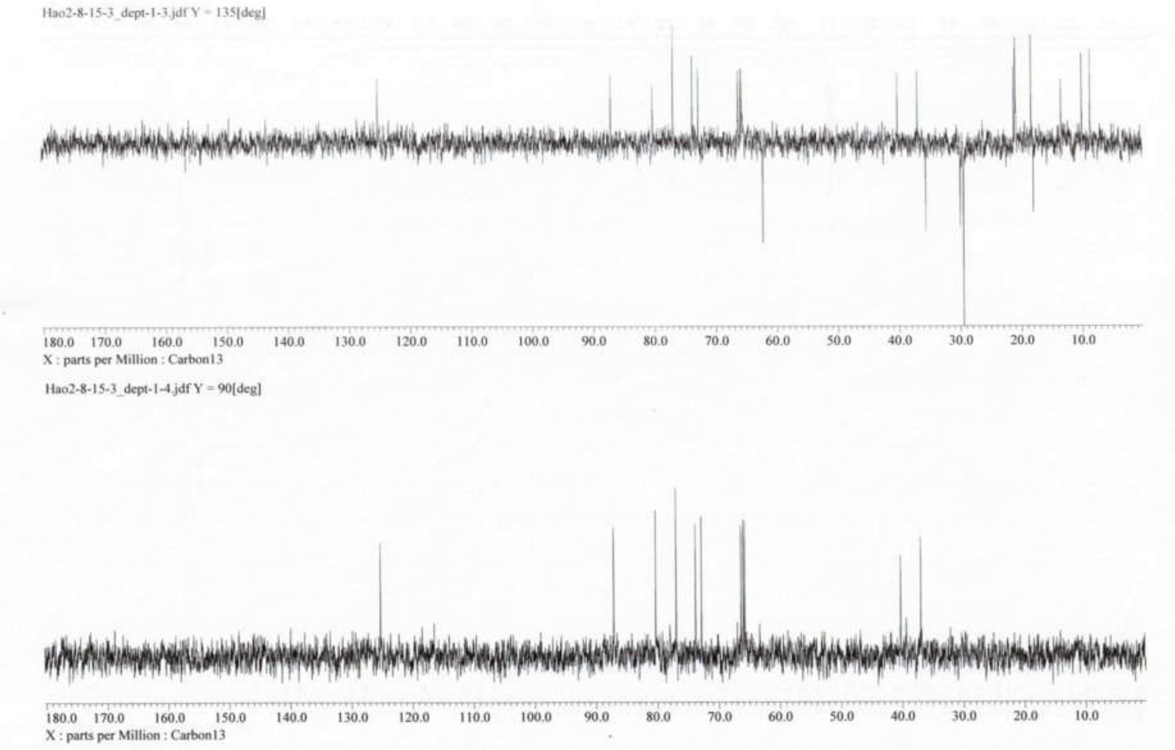

Figure S6. DEPT spectrum (100 MHz) of compound **1** in $CDCl_3$.

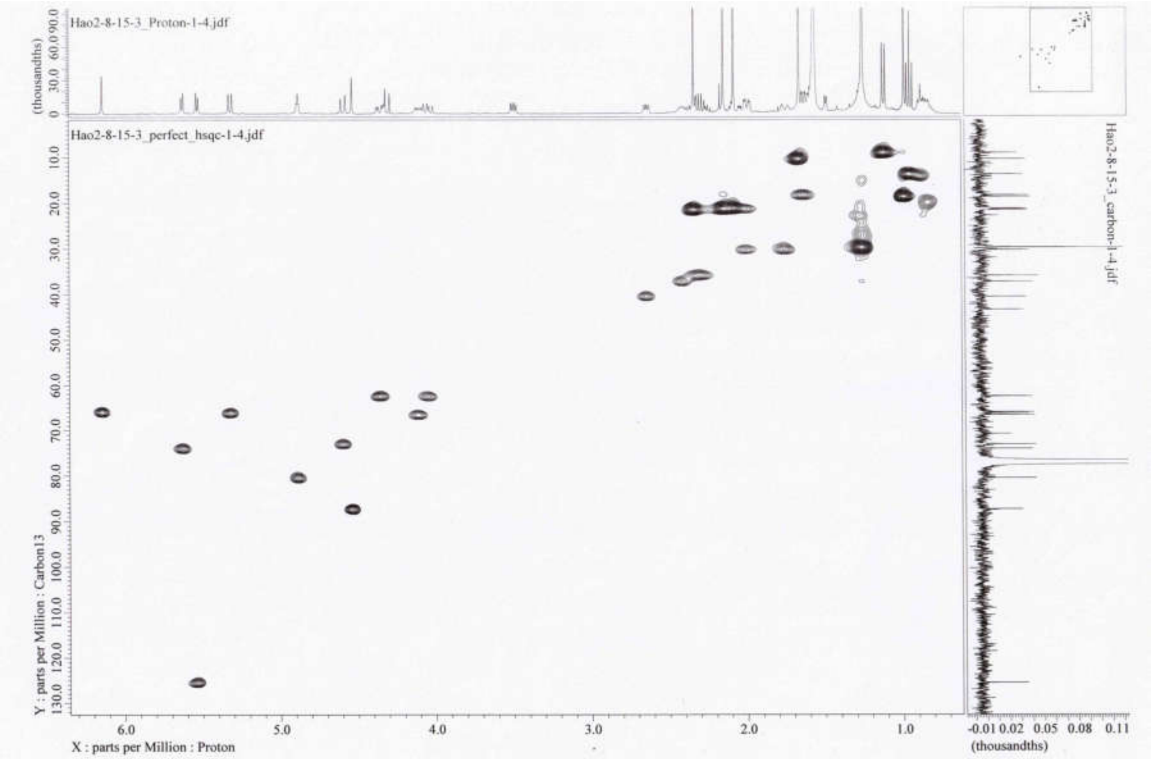

Figure S7. HSQC spectrum of compound **1** in $CDCl_3$.

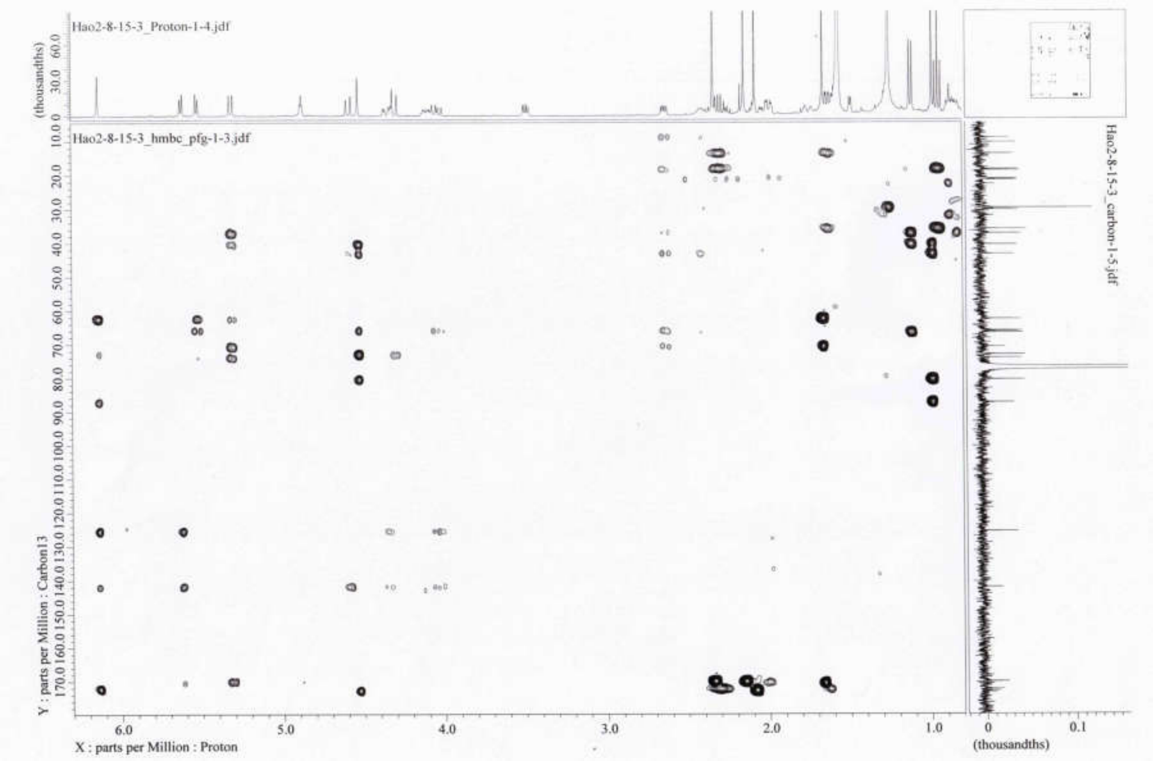

Figure S8. HMBC spectrum of compound **1** in $CDCl_3$.

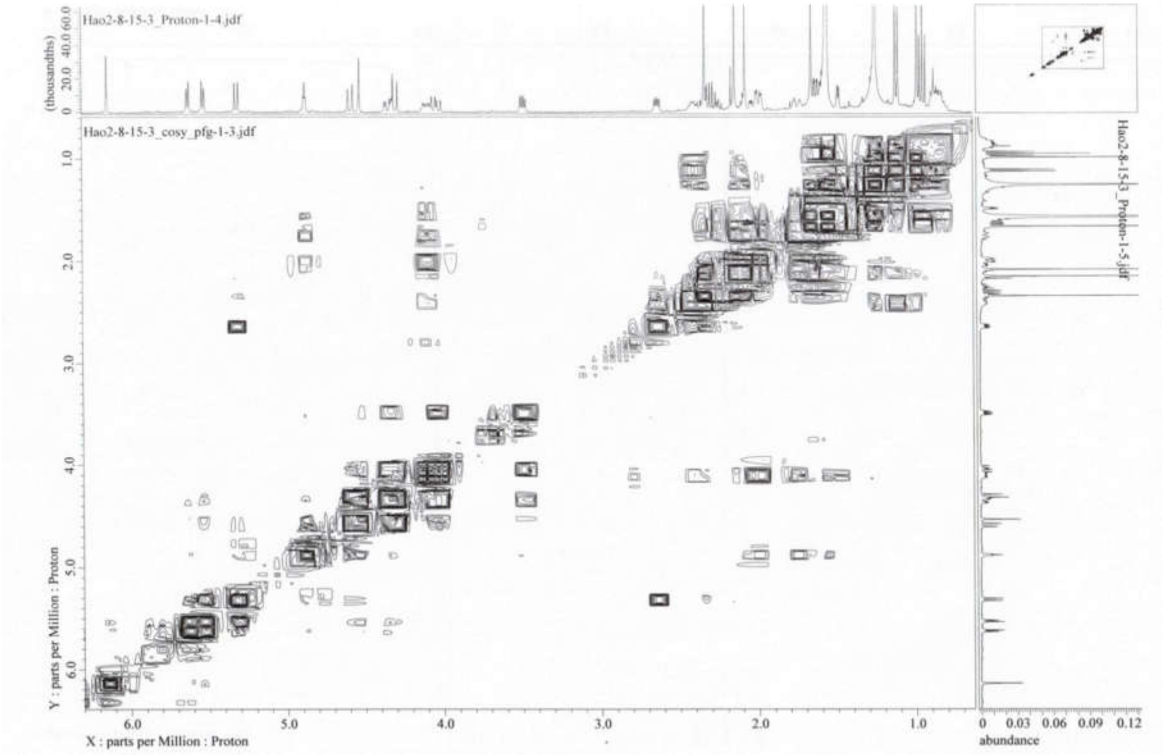

Figure S9. ^1H-^1H COSY spectrum of compound **1** in $CDCl_3$.

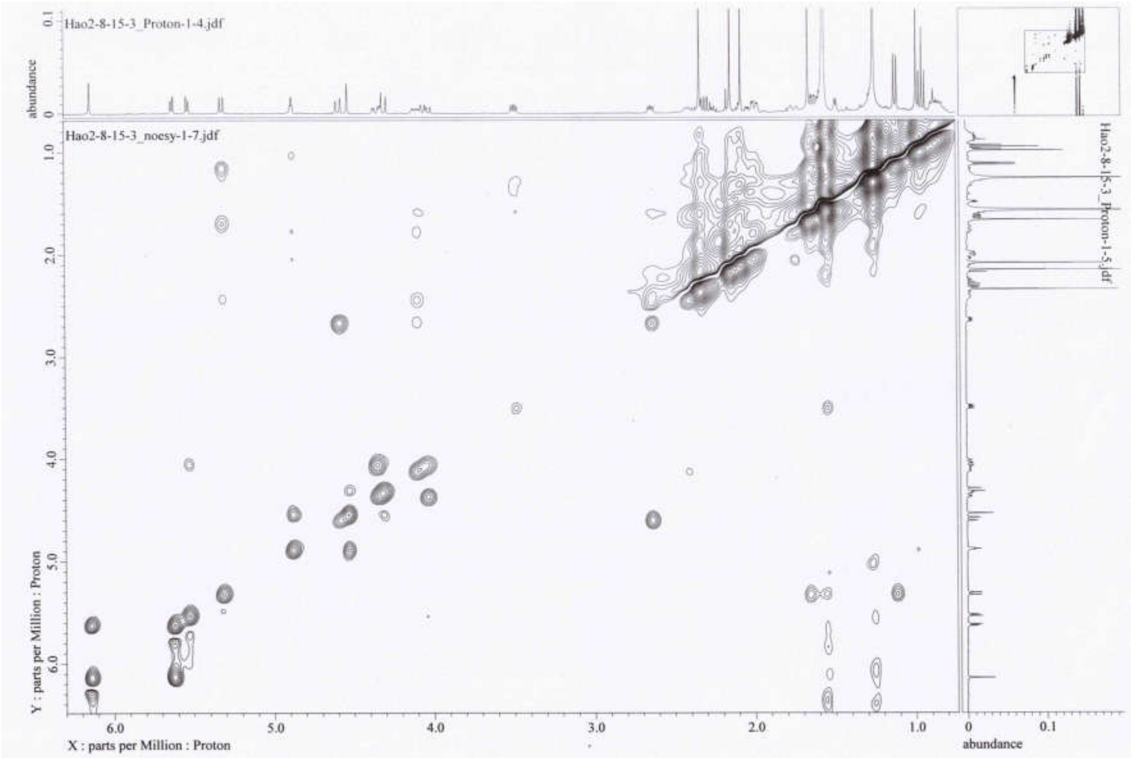

Figure S10. NOESY spectrum of compound **1** in $CDCl_3$.

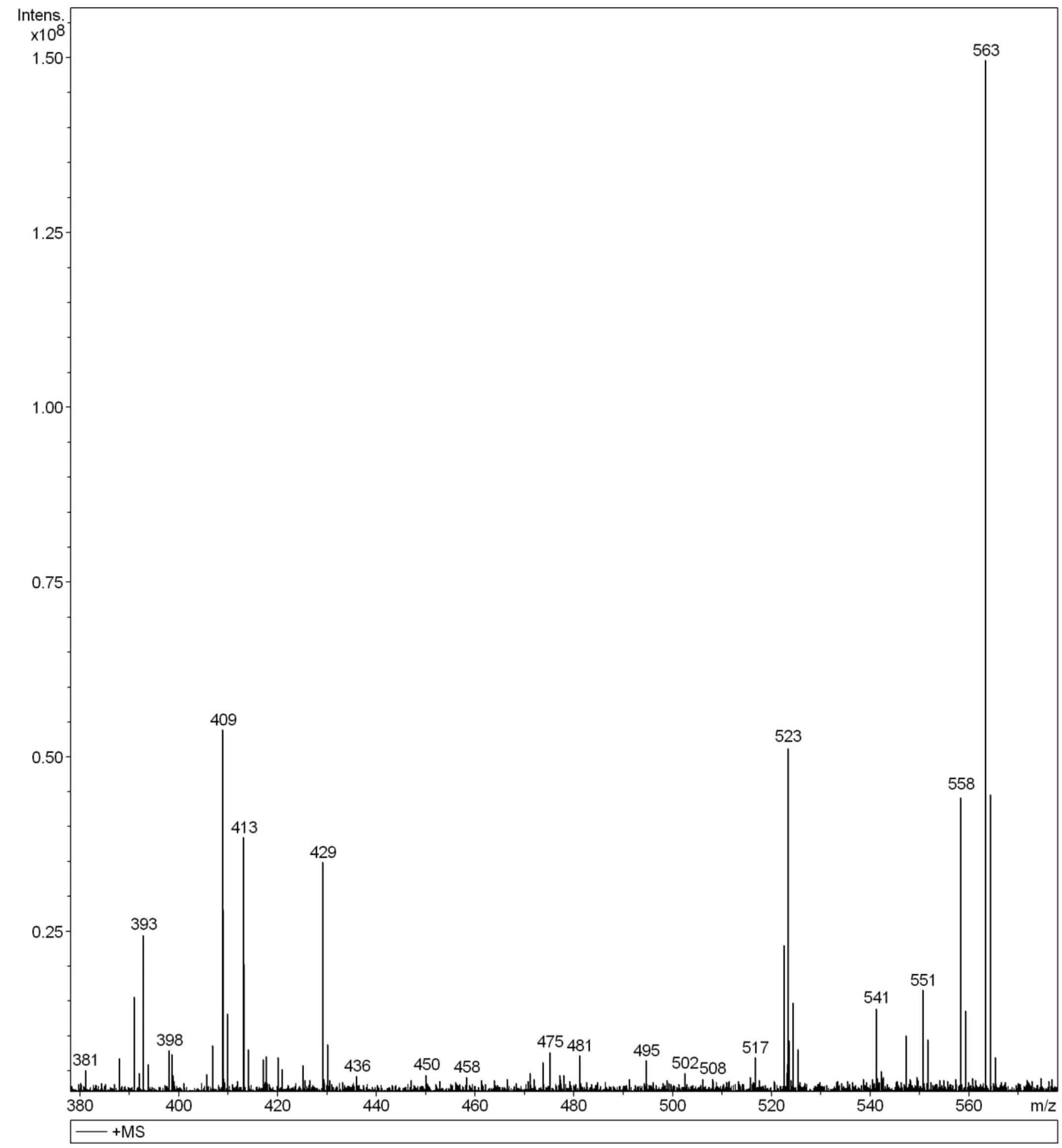

Figure S11. ESIMS spectrum of compound **2.**

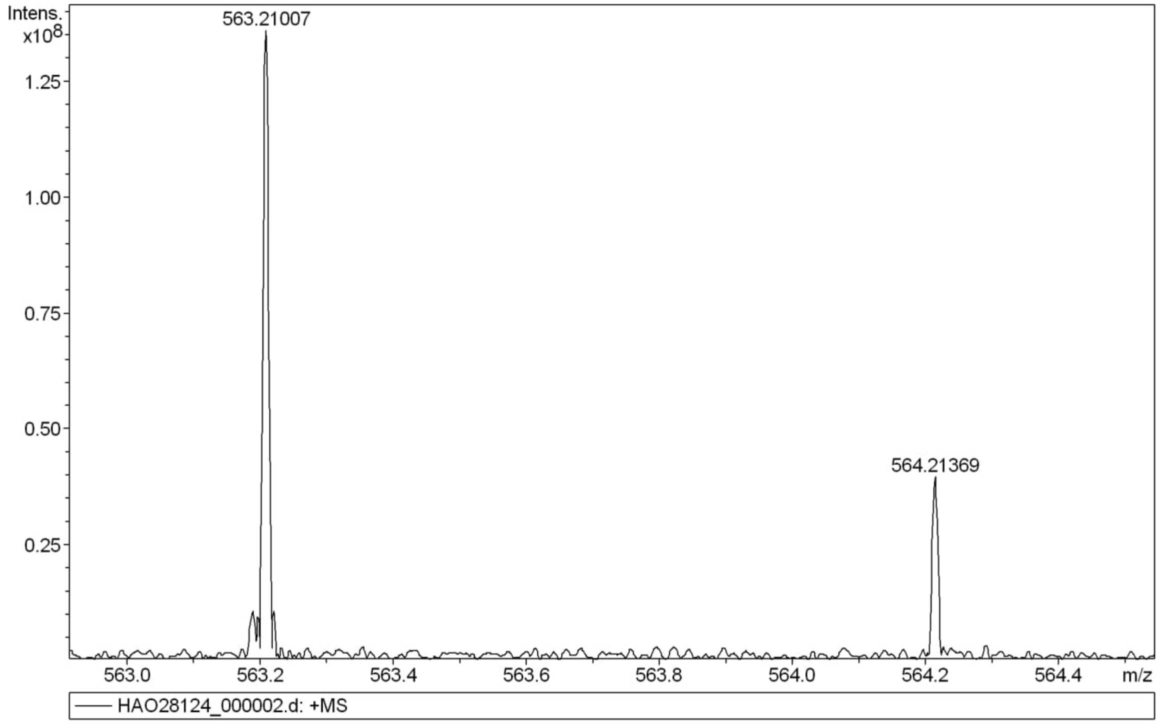

Meas. m/z	#	Formula	Score	m/z	err [mDa]	err [ppm]	mSigma	rdb	e⁻ Conf	N-Rule
563.21007	1	C 26 H 36 Na O 12	100.00	563.20990	-0.17	-0.30	4.0	8.5	even	ok

Figure S12. HRESIMS spectrum of compound **2.**

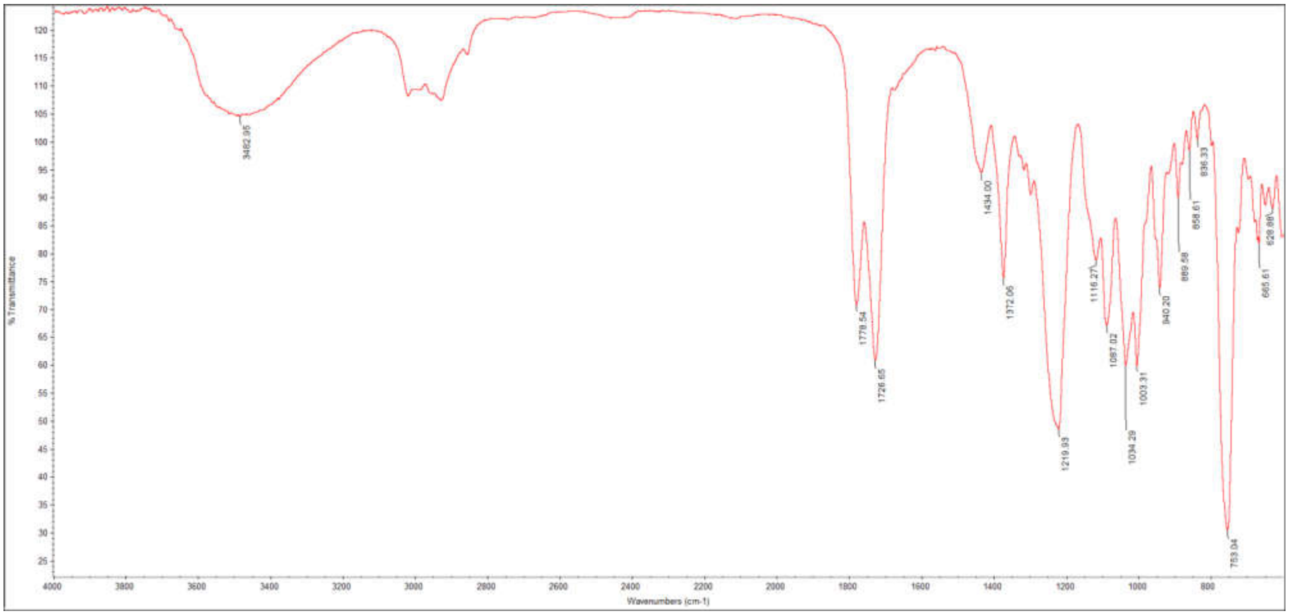

Figure S13. IR spectrum of compound **2.**

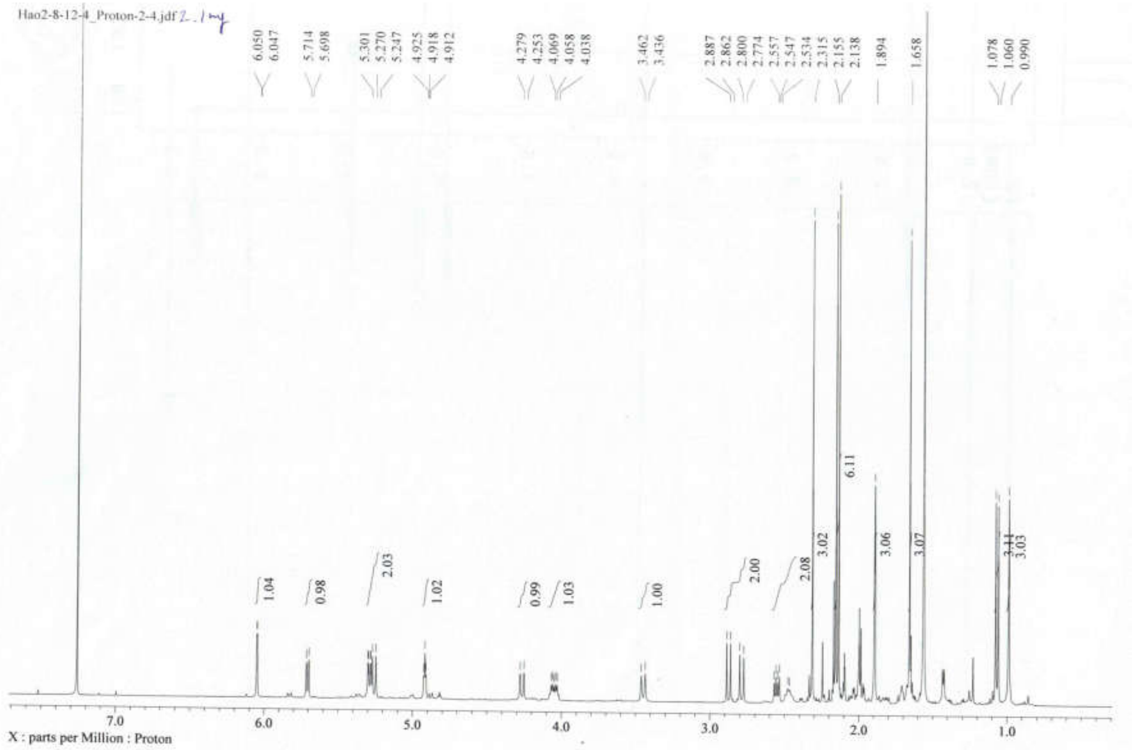

Figure S14. 1H NMR spectrum (400 MHz) of compound 2 in CDCl3.

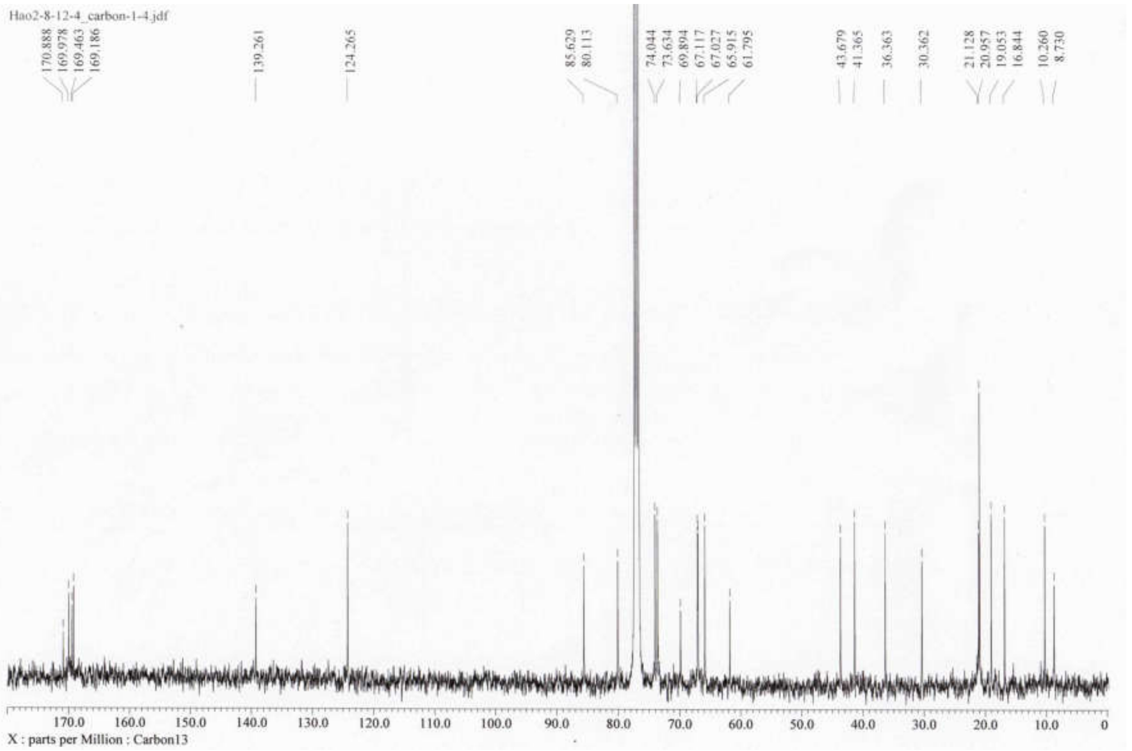

Figure S15. ^{13}C NMR spectrum (100 MHz) of compound **2** in $CDCl_3$.

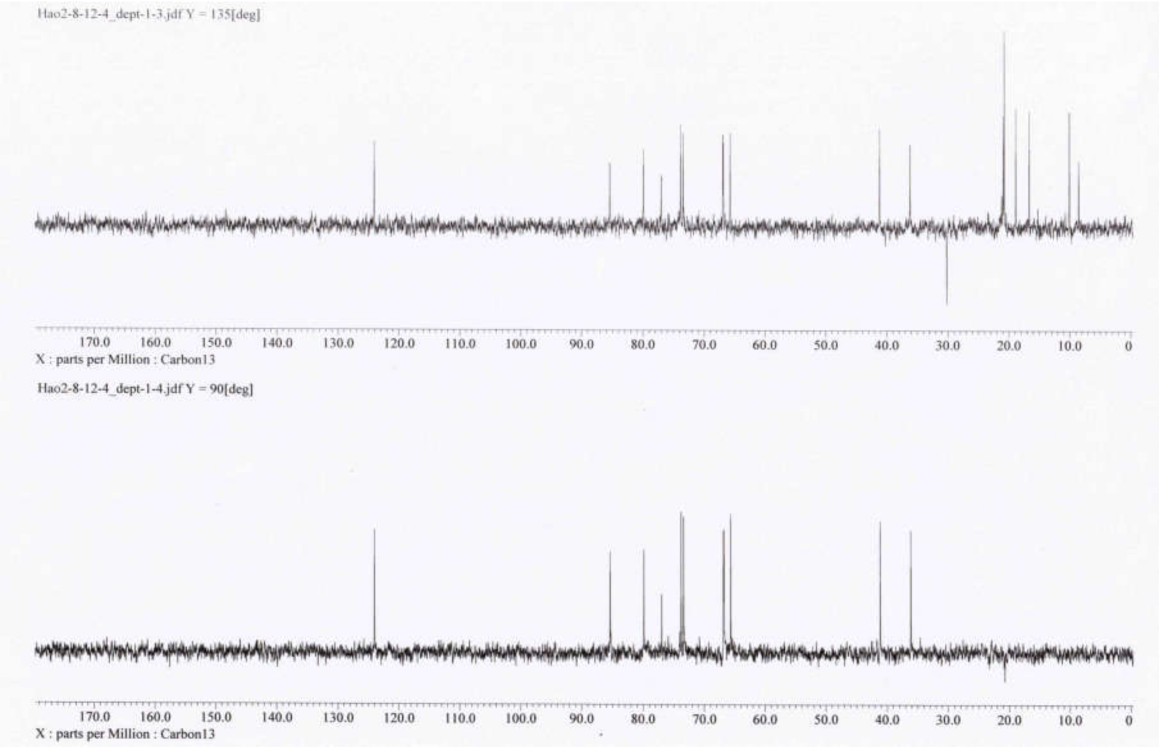

Figure S16. DEPT spectrum (100 MHz) of compound **2** in $CDCl_3$.

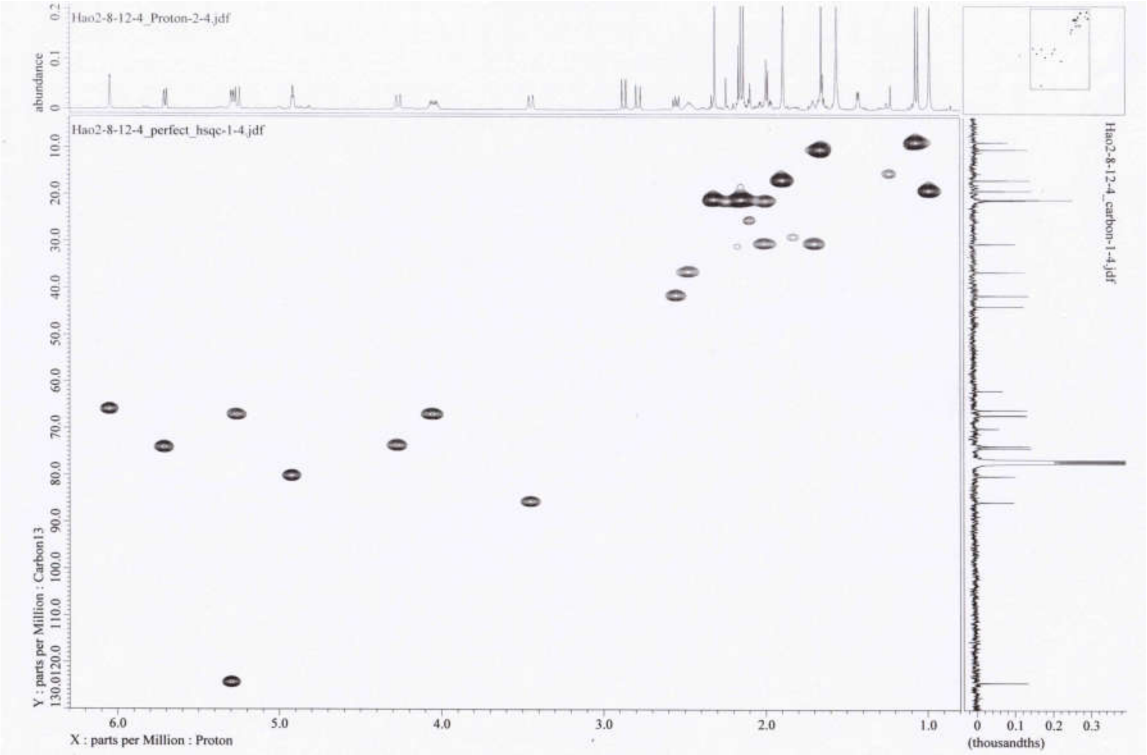

Figure S17. HSQC spectrum of compound **2** in $CDCl_3$.

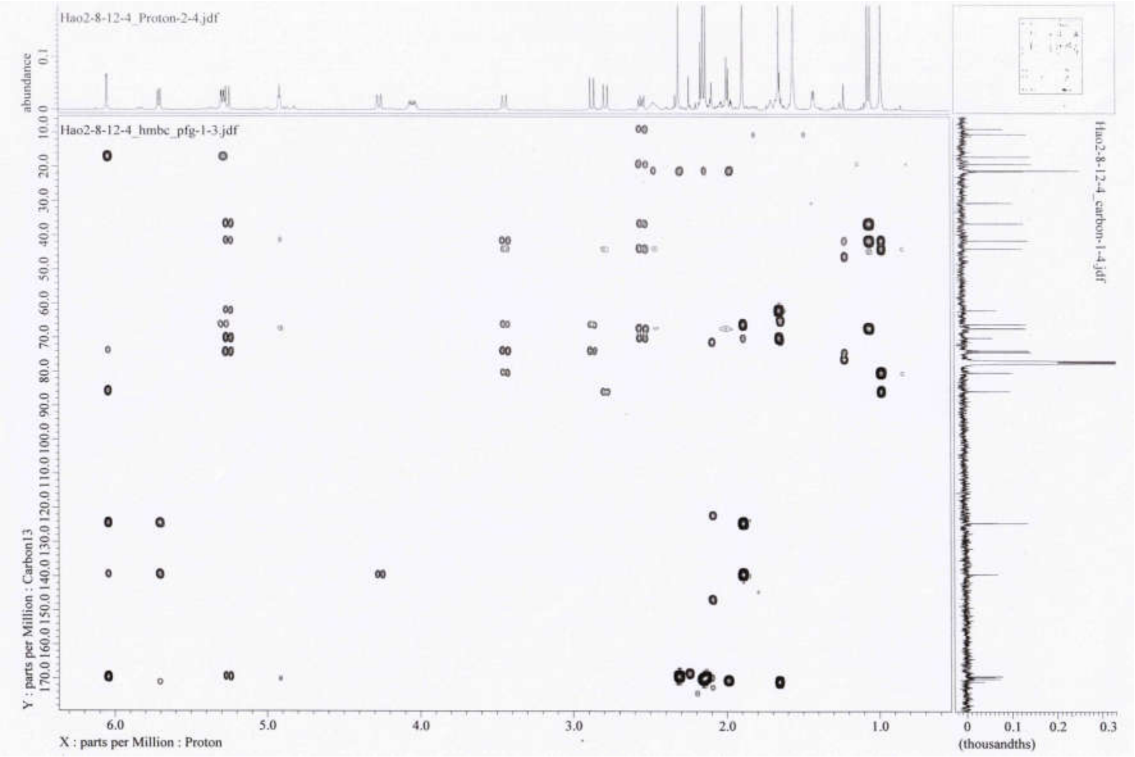

Figure S18. HMBC spectrum of compound **2** in $CDCl_3$.

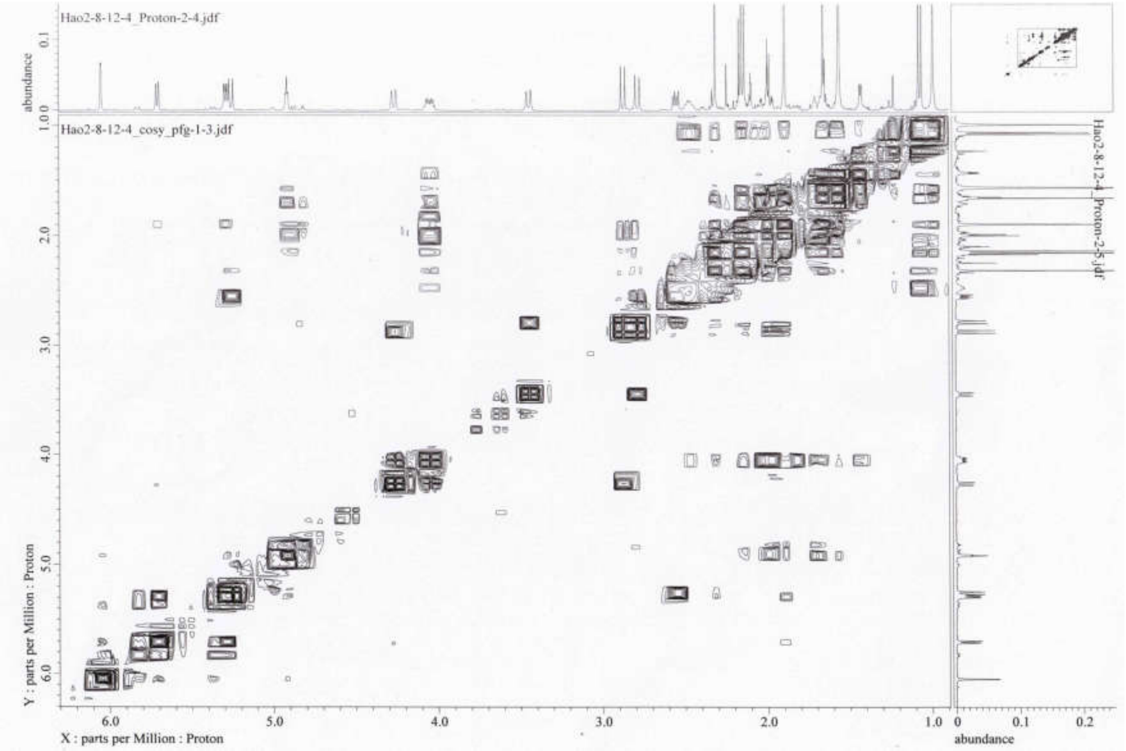

Figure S19. ^1H-^1H COSY spectrum of compound **2** in $CDCl_3$.

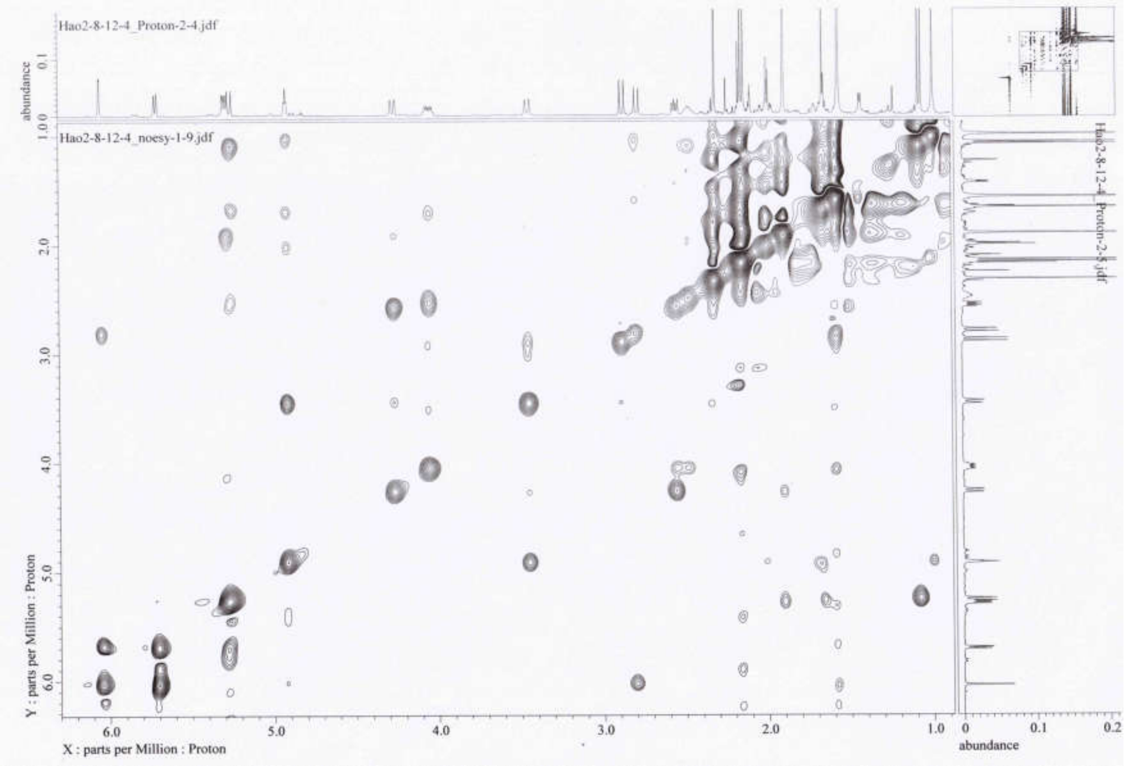

Figure S20. NOESY spectrum of compound **2** in $CDCl_3$.

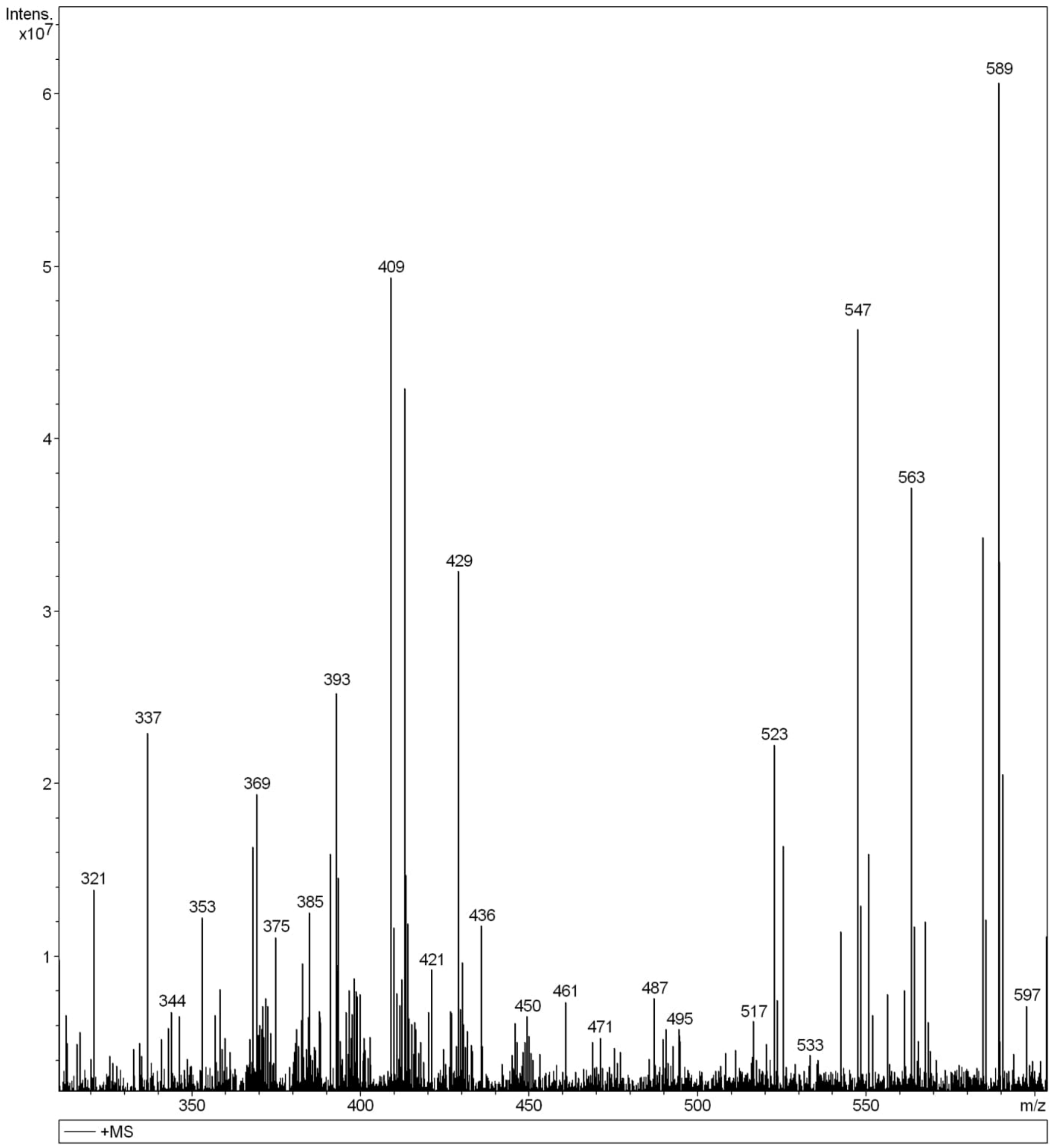

Figure S21. ESIMS spectrum of compound **3.**

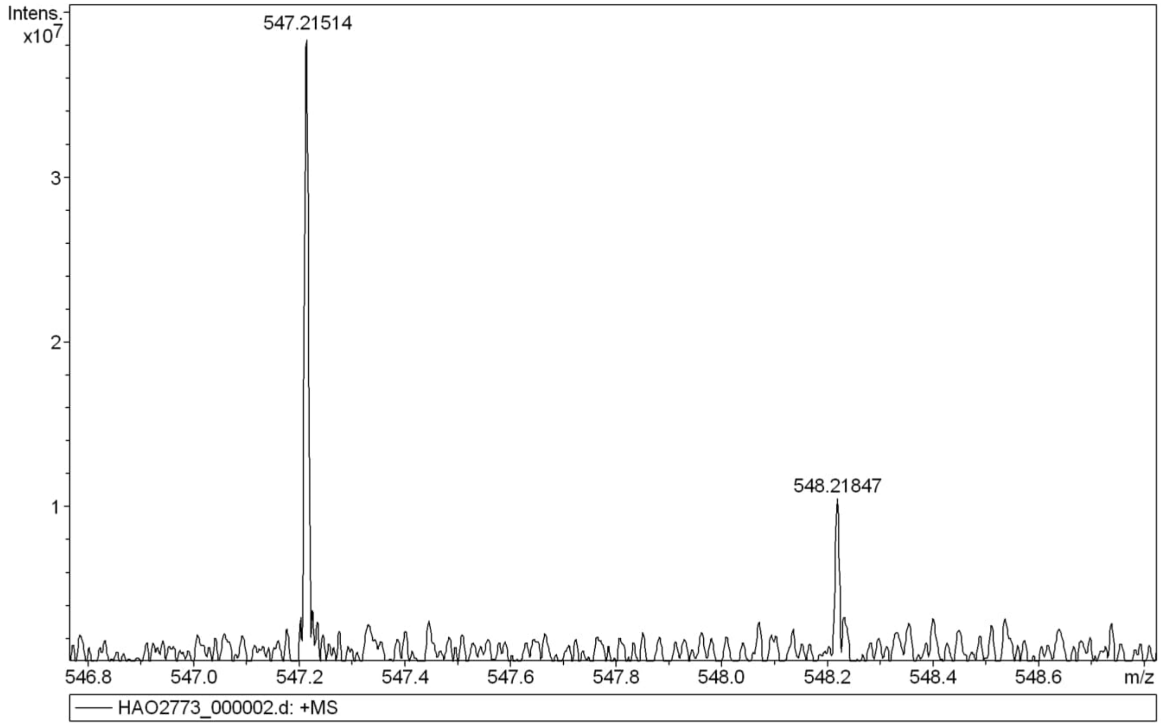

Meas. m/z	#	Formula	Score	m/z	err [mDa]	err [ppm]	mSigma	rdb	e⁻ Conf	N-Rule
547.21514	1	C 26 H 36 Na O 11	100.00	547.21498	-0.16	-0.30	32.8	8.5	even	ok

Figure S22. HRESIMS spectrum of compound **3.**

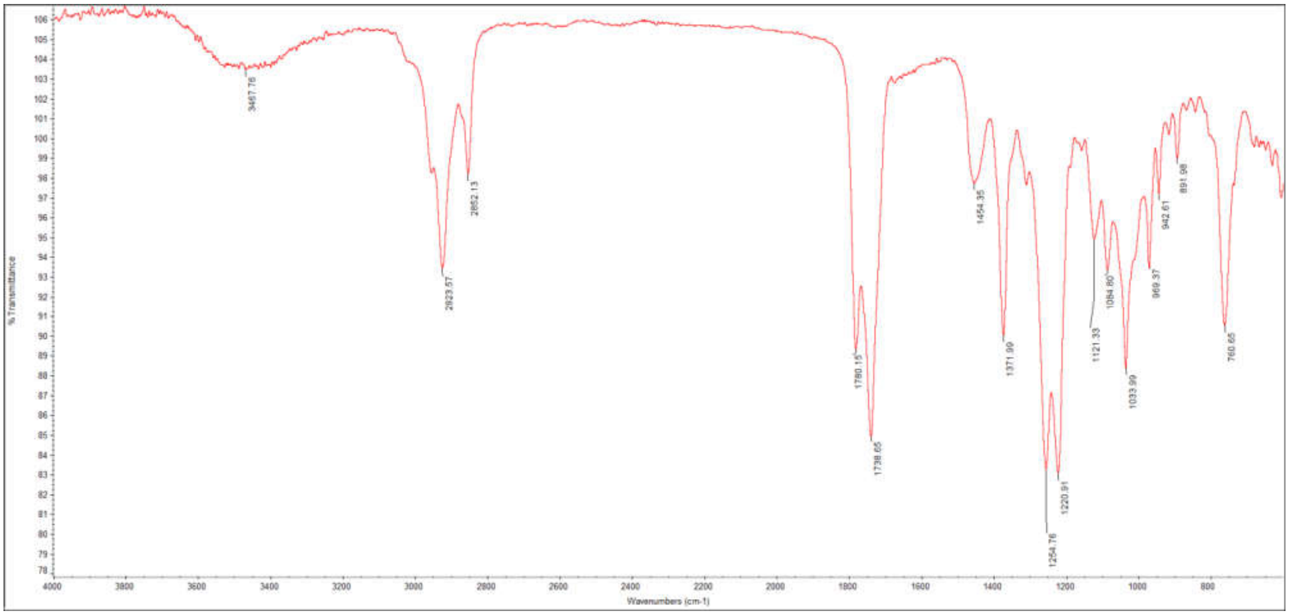

Figure S23. IR spectrum of compound **3.**

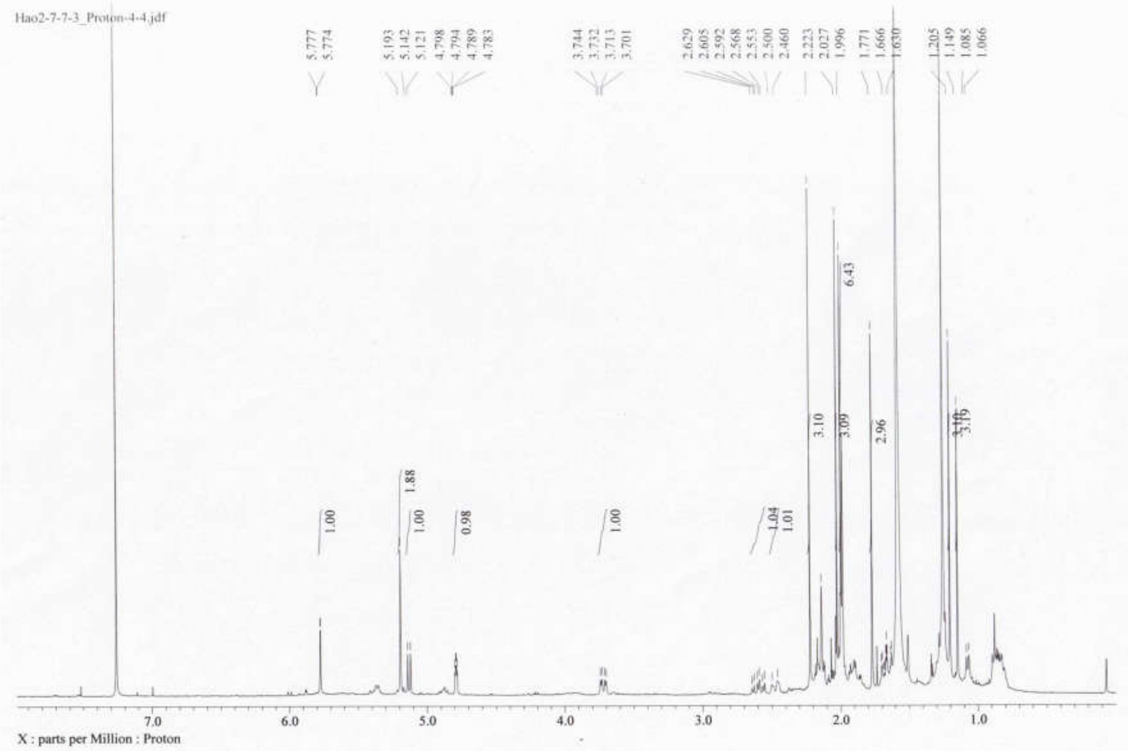

Figure S24. ^{1}H NMR spectrum (400 MHz) of compound **3** in $CDCl_3$.

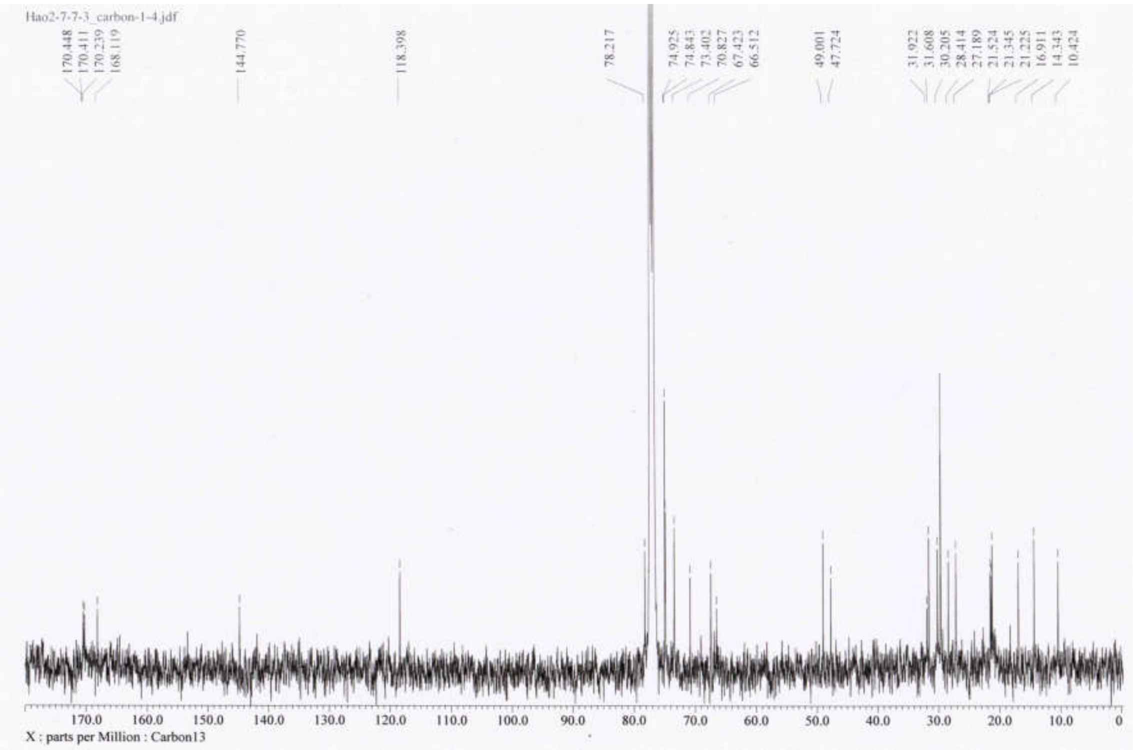

Figure S25. ^{13}C NMR spectrum (100 MHz) of compound **3** in $CDCl_3$.

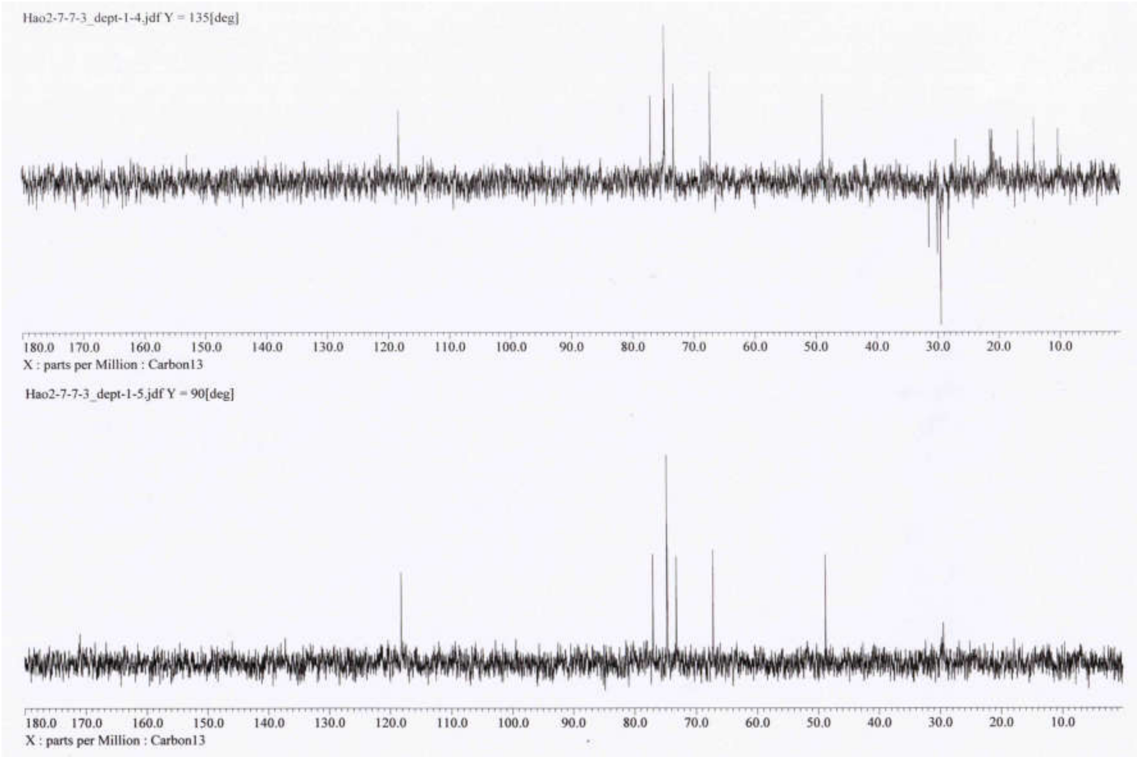

Figure S26. DEPT spectrum (100 MHz) of compound **3** in $CDCl_3$.

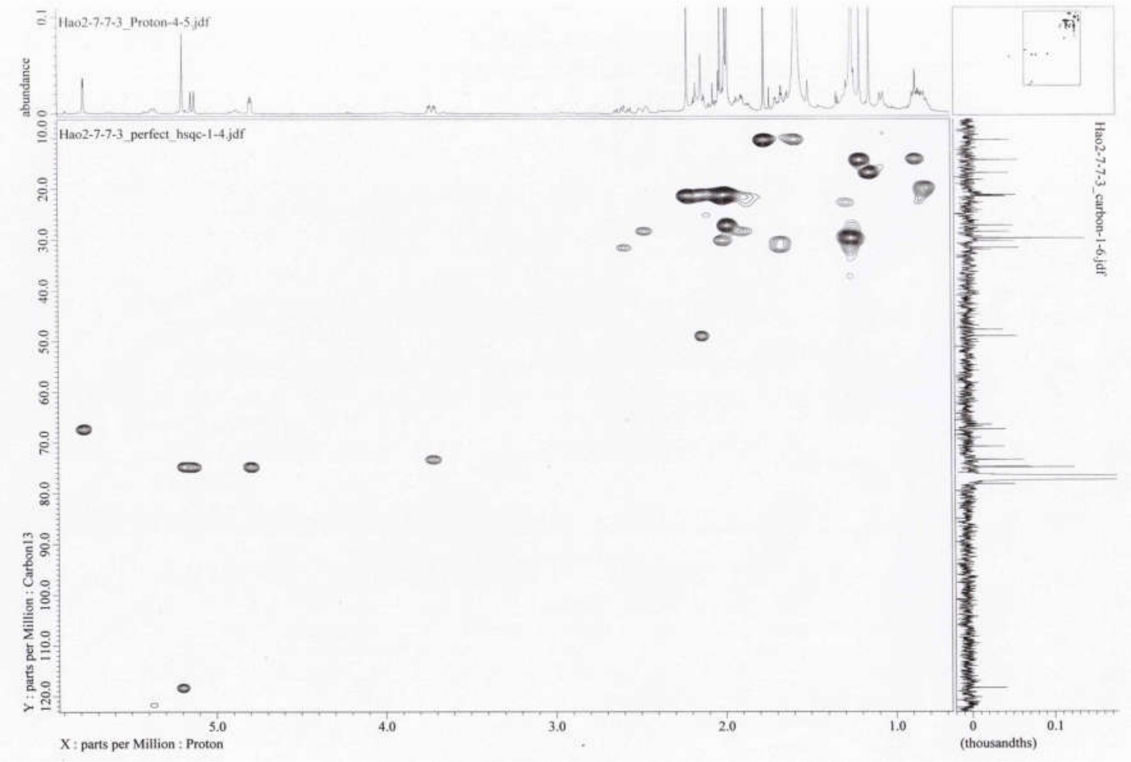

Figure 27. HSQC spectrum of compound **3** in $CDCl_3$.

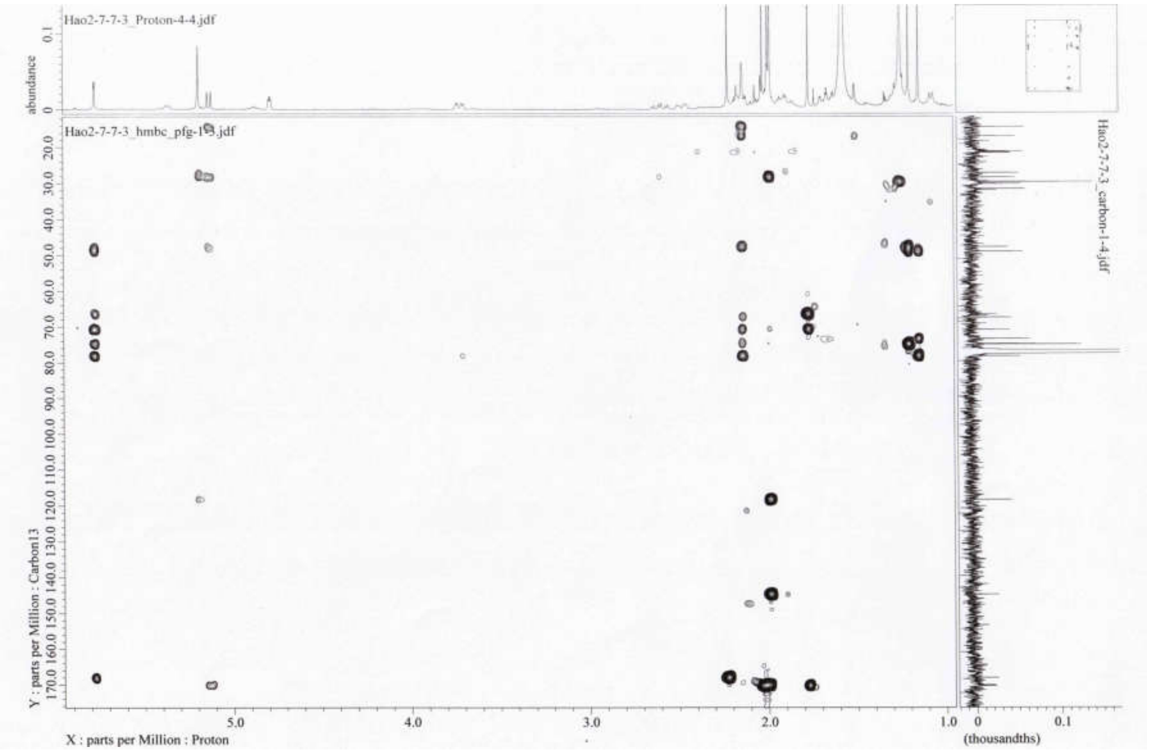

Figure S28. HMBC spectrum of compound **3** in $CDCl_3$.

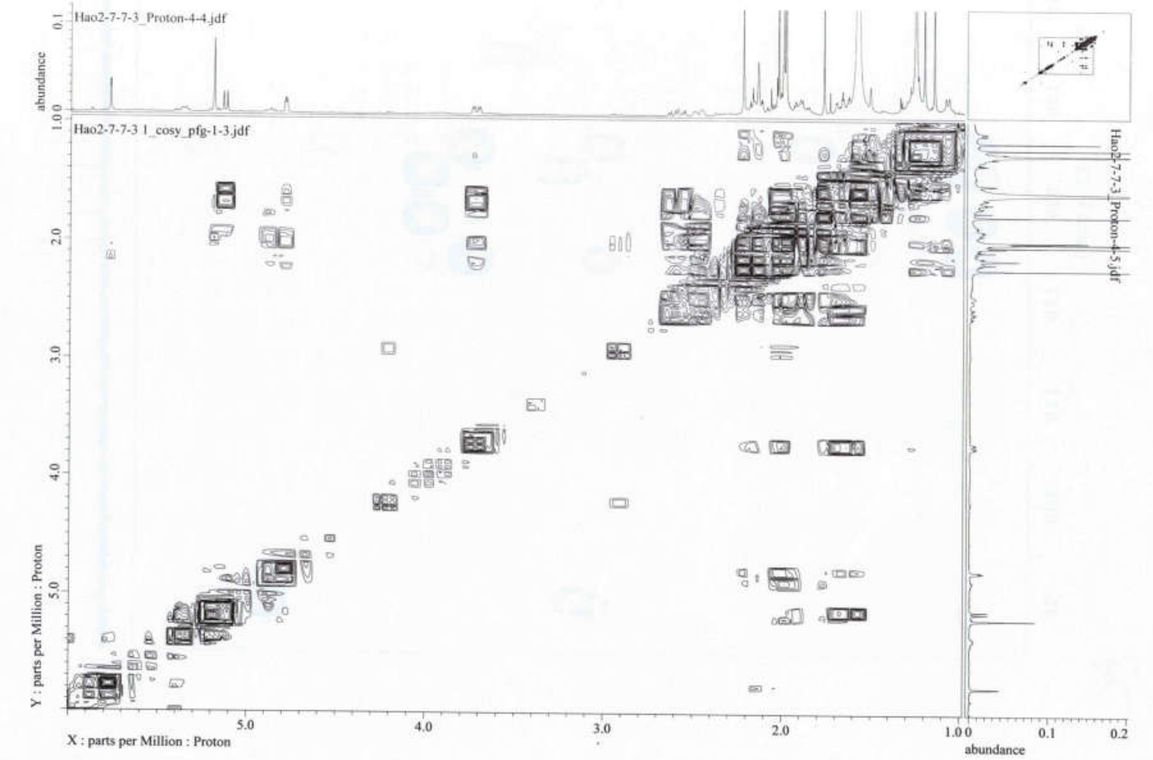

Figure 29. ^1H-^1H COSY spectrum of compound **3** in $CDCl_3$.

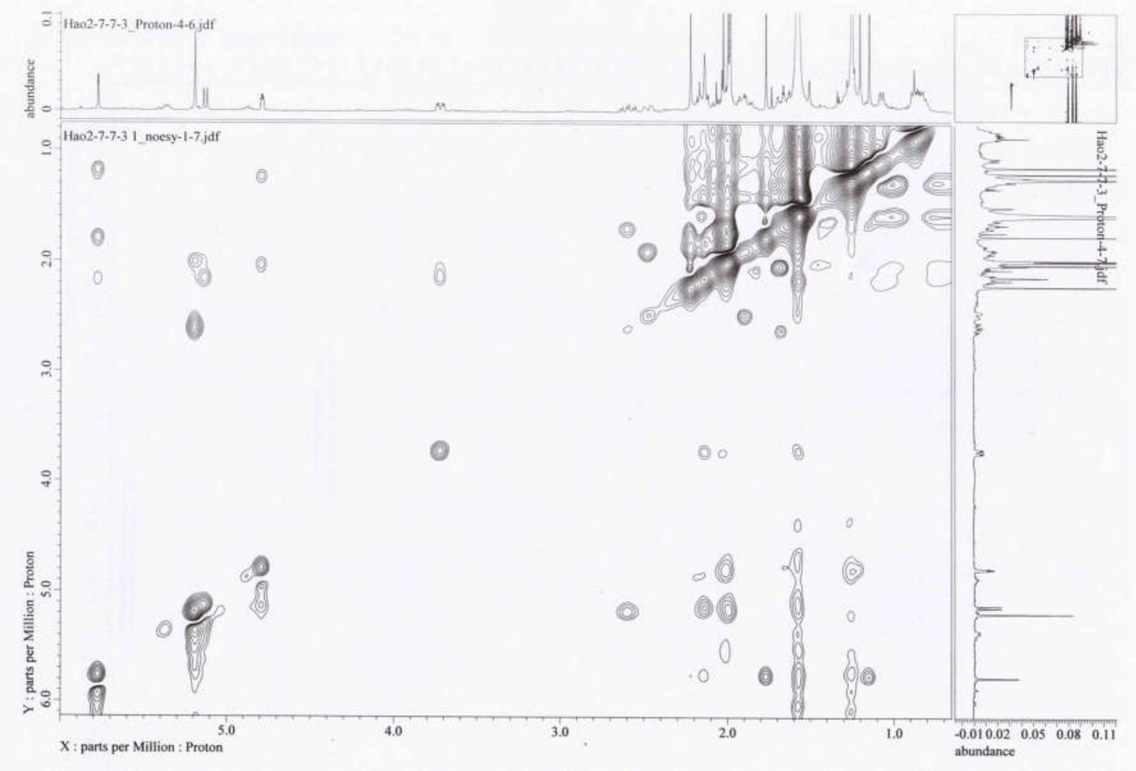

Figure S30. NOESY spectrum of compound **3** in $CDCl_3$.
